# Global Analysis of *Arabidopsis*/Downy Mildew Interactions Reveals Prevalence of Incomplete Resistance and Rapid Evolution of Pathogen Recognition

**DOI:** 10.1371/journal.pone.0028765

**Published:** 2011-12-14

**Authors:** Ksenia V. Krasileva, Connie Zheng, Lauriebeth Leonelli, Sandra Goritschnig, Douglas Dahlbeck, Brian J. Staskawicz

**Affiliations:** Department of Plant and Microbial Biology, University of California, Berkeley, California, United States of America; University of California, United States of America

## Abstract

Interactions between *Arabidopsis thaliana* and its native obligate oomycete pathogen *Hyaloperonospora arabidopsidis (Hpa)* represent a model system to study evolution of natural variation in a host/pathogen interaction. Both *Arabidopsis* and *Hpa* genomes are sequenced and collections of different sub-species are available. We analyzed ∼400 interactions between different *Arabidopsis* accessions and five strains of *Hpa*. We examined the pathogen's overall ability to reproduce on a given host, and performed detailed cytological staining to assay for pathogen growth and hypersensitive cell death response in the host. We demonstrate that intermediate levels of resistance are prevalent among *Arabidopsis* populations and correlate strongly with host developmental stage. In addition to looking at plant responses to challenge by whole pathogen inoculations, we investigated the *Arabidopsis* resistance attributed to recognition of the individual *Hpa* effectors, ATR1 and ATR13. Our results suggest that recognition of these effectors is evolutionarily dynamic and does not form a single clade in overall *Arabidopsis* phylogeny for either effector. Furthermore, we show that the ultimate outcome of the interactions can be modified by the pathogen, despite a defined gene-for-gene resistance in the host. These data indicate that the outcome of disease and disease resistance depends on genome-for-genome interactions between the host and its pathogen, rather than single gene pairs as thought previously.

## Introduction

*Hyaloperonospora arabidopsidis* (*Hpa*, formerly known as *Peronospora parasitica*) is a native downy mildew pathogen of the plant model organism *Arabidopsis thaliana* [1]–[2]. *Hpa* is an obligate biotrophic pathogen, propagating to a new host by means of small asexual conidiospores that form on sporangiophores emerging from the plant leaf surface after successful colonization of plant leaf tissues. Occasionally, sexual oospores form inside the plant, generating genetic diversity for the pathogen [1]. Host plant defense responses are induced shortly after the pathogen starts to grow. A visible hallmark of plant defense is the induction of the hypersensitive cell-death response [1]. Genetic analyses of *Arabidopsis* disease resistance to *Hpa* have identified several dozens disease resistance genes [3], [4], [5], [6], [7], while genetic and bioinformatic analyses in *Hpa* have led to the identification of several confirmed effectors [8], [9], [10], and the prediction of 130–150 putative effector genes [11], [12]. The obligate nature of the interactions between *Hpa* and *Arabidopsis* has brought evolutionary pressure on both the pathogen and the host. Many *Hpa* effectors have been shown to be under the pressure of strong positive selection [8], [11]. Similar evolutionary patterns have been observed for many *Arabidopsis* disease resistance genes, which occur in multiple copies at complex genetic loci [4], [8]. Understanding the genetic and phenotypic diversity of *Arabidopsis/Hpa* interactions can provide valuable insight into the co-evolution between obligate eukaryotic pathogens and their respective hosts.

Current genome projects aim to sequence and characterize more than a thousand *A. thaliana* sub-species, called ecotypes or accessions [13], [14]. A set of 95 *Arabidopsis* accessions from worldwide locations (the Nordborg collection) has been extensively characterized based on small nucleotide polymorphisms and genome-wide association analyses of numerous phenotypes including flowering time and resistance to bacterial pathogens [13], [15]. Similarly, several *Hpa* strains collected in their natural habitat are available [3], and the genome sequence of *Hpa* strain Emoy2 has recently been published [12]. Furthermore, the number of complete genome sequences of *Arabidopsis* sub-species and *Hpa* strains is rapidly increasing due to development of high-throughput sequencing technologies. Understanding the significance of genetic variation within host/pathogen interactions requires knowledge of the corresponding phenotypic variation gained through careful characterization of interactions between *Arabidopsis* and *Hpa*.

There are two approaches to measure a pathogen's interaction with the host: pathogen transmissibility (the basic ability to complete its life cycle and propagate its progeny), and disease severity (the amount of damage caused to the host due to the pathogen's activities or induction of host immune responses). Two previous studies have addressed *Arabidopsis/Hpa* interactions on a population level. Eric Holub observed infected *Arabidopsis* cotyledons and developed an excellent qualitative scoring system based on visual estimation of the amount and intensity of plant cell death, which he applied to a population of *Arabidopsis* accessions collected in the United Kingdom [16]. In addition, a recent study analyzed the Nordborg collection and made observations of infected true leaves, ranking them as susceptible, resistant or intermediate based solely on the presence of pathogen asexual spores [17]. However, a report observing the interaction between *Arabidopsis* accession Col-0 and *Hpa* strain Emco5 showed that this interaction was controlled by host development; in this particular case, the pathogen was fully virulent on *Arabidopsis* cotyledons, but failed to reproduce on true leaves [18]. Moreover, the amount of pathogen growth and plant cell death was substantially different between cotyledons and true leaves [18]. Therefore, we undertook a more comprehensive study to address the prevalence of developmental control in this host-pathogen interaction.

On the molecular level, much effort has been put to investigate two known pathogen-derived effectors, *Arabidopsis thaliana recognized 1* (*ATR1*) and *ATR13*, and their cognate resistance (*R*) genes *RPP1* and *RPP13* [8], [9], [19], [20], [21], [22], [23]. However, the relative contributions of these two effectors on global *Arabidopsis/Hpa* interactions are not well understood. Even more interesting are the open questions concerning the evolution of oomycete effector recognition by the host. A study of *Arabidopsis* accessions from the United Kingdom shows that recognition of ATR13 in *Arabidopsis* is genetically complex, and can be mapped to two independent *Arabidopsis* loci [24]. Similarly, the RPP13 locus, originally identified to be responsible for recognition of ATR13, can recognize a different oomycete effector in some accessions [24]. Our recent studies on ATR1 suggest that its recognition in two *Arabidopsis* accessions could have evolved independently [25]. Results from both of these studies challenge the simplicity of gene-for-gene interactions between host and pathogen, suggesting that a more global analysis of effector/*R* gene interactions is needed in order to formulate new hypotheses. Development of a surrogate oomycete effector delivery based on the bacterial Type III Secretion System (TTSS) has enabled us to introduce individual oomycete effectors into the host. ATR1 and ATR13 delivered by TTSS induce resistance that is able to suppress growth of pathogenic bacteria in plants containing the cognate R genes, *RPP1* and *RPP13* [19], [20]. Therefore, standard bacterial growth curves can be used as a quantitative measure for the resistance conferred by a particular *Hpa* effector. This surrogate system overcomes the challenges of working with an obligate, genetically intractable pathogen and provided us with a rapid quantitative method to screen *Arabidopsis* accessions with known *Hpa* effectors.

In this study, we present a detailed analysis of ∼400 *Arabidopsis/Hpa* interactions using a subset of accessions from the Nordborg collection and five *Hpa* strains isolated in the United Kingdom. Examining each genotype-by-genotype interaction, we recorded the ability of the pathogen to produce asexual spores, as well as the amount of pathogen growth and the extent of plant cell death. As a result, we developed a quantitative scoring system to describe five types of observed *Arabidopsis/Hpa* interactions. We recorded our observations on both *Arabidopsis* cotyledons and true leaves, observing prevalence of incomplete resistance and a strong dependence on host developmental stage. Finally, we used the TTSS delivery system to deliver several alleles of the *Hpa* effectors ATR1 and ATR13 into the *Arabidopsis* accessions. Interestingly, ATR1 and ATR13-specified immunity is rare among *Arabidopsis* accessions and does not correlate with overall genome genealogy. In addition, examination of the plant response to individual effectors versus whole *Hpa* pathogen infection revealed a situation in which a functional effector-triggered immunity is suppressed by the pathogen. Overall, this study provides an extensive phenotypic library of *Arabidopsis/Hpa* interactions. Most importantly, our data shows the need to move beyond the gene-for-gene hypothesis to the understanding of co-evolution and interactions of multiple genomic components in host and pathogen.

## Results

### Race-specific interactions between *Arabidopsis* and *Hpa* show little correlation to overall *Arabidopsis* genealogy

**Figure 1:**
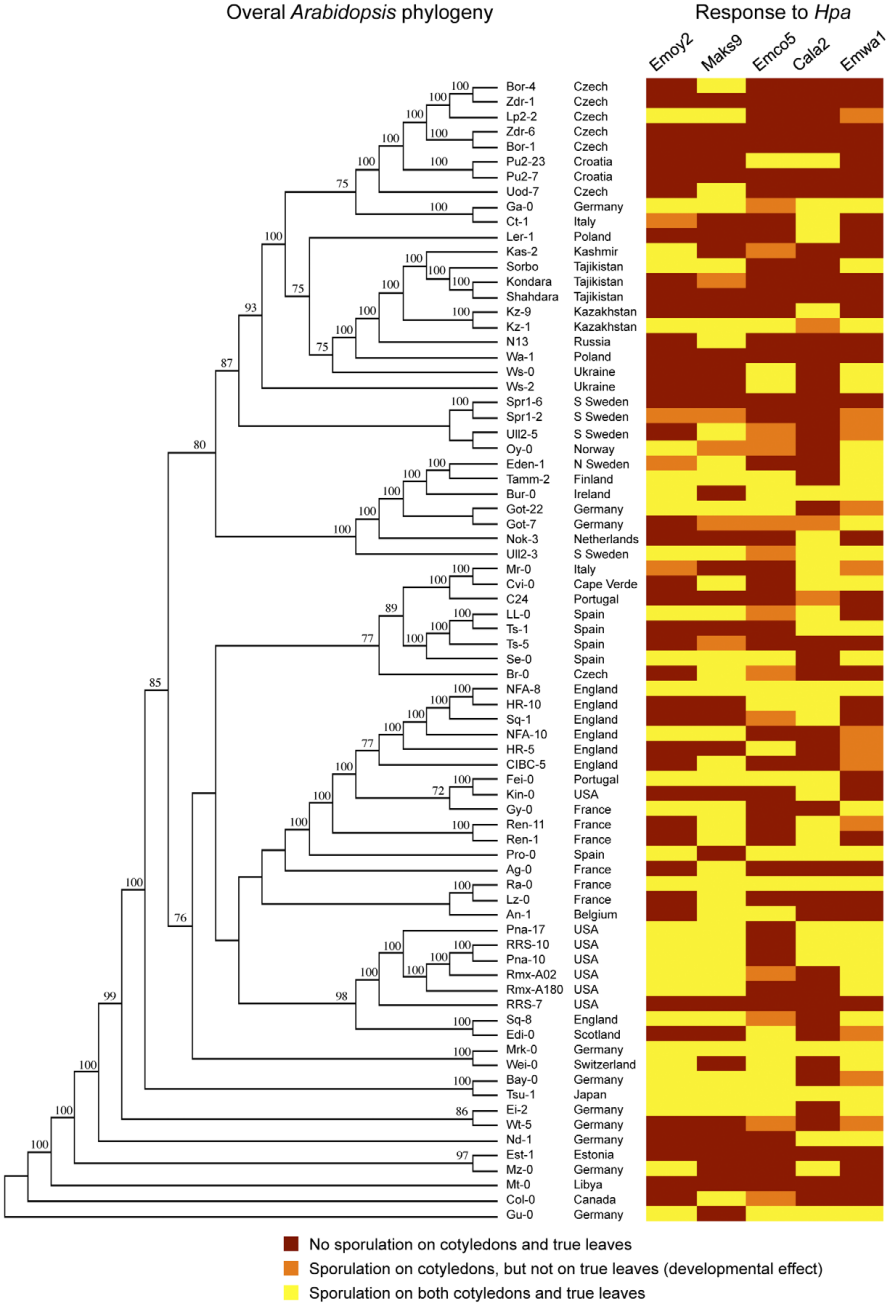
Resistance to *Hpa* compared with overall *Arabidopsis* phylogeny. The phylogenetic tree on the left represents a reconstruction of the overall genealogy of 72 *Arabidopsis* accessions derived from 205k genome-wide small nucleotide polymorphism data published previously [13]. Bootstrap values (>70) are displayed on the branches of the tree. The *Hpa* sporulation data obtained in this study is displayed on the right and color-coded according to the ability of the pathogen to produce sporangiophores: red – no sporulation, orange – sporulation only on cotyledons, but not on true leaves, yellow – sporulation on both cotyledons and true leaves.

We examined interactions between 83 accessions of *Arabidopsis thaliana*, collected from diverse locations around the world [15] (stock numbers are listed in Table S1), and five strains of *Hyaloperonospora arabidopsidis* (*Hpa*) originally isolated in the United Kingdom [3]. *Hpa* growth was macroscopically assessed by scoring for the presence of sporangiophores emerging from the plant cotyledons and true leaves. Plants within a single accession did not exhibit substantial variation in response to a given *Hpa* strain. Global *Arabidopsis* susceptibility to *Hpa*, depending on which strain was applied, ranged from 42% to 56% on cotyledons and from 27% to 50% on true leaves (**Table 1**). The *Hpa* strain Emco5 was least virulent on true leaves, producing asexual spores only on 27% of the examined accessions (**Table 1**), similar to what has been previously reported [17]. However, our analysis indicates that *Hpa* Emco5 successfully colonized 42% of *Arabidopsis* cotyledons, comparable to other strains used in this study (**Table 1**). The overall pattern of disease resistance or disease susceptibility showed no clear correlation with geographic origin of *Arabidopsis* accessions. To examine whether susceptibility or resistance to *Hpa* strains correlated with overall phylogenetic relatedness among *Arabidopsis* accessions, we re-constructed an *Arabidopsis* genealogy (**Figure 1**) based on the available 205K SNP data [13]. Interestingly, we observed little correlation of disease resistance with the overall genome-wide relatedness of *Arabidopsis* accessions (**Figure 1**), suggesting complex evolutionary interactions between the pathogen and its host.

**Table 1:** Percentage of *Arabidopsis* accessions supporting *Hpa* sporulation.

	*H. arabidopsidis* strain				
	Emoy2	Maks9	Emco5	Cala2	Emwa1
Cotyledons	48.19%	57.83%	42.17%	45.78%	55.42%
True Leaves	43.37%	51.81%	26.51%	40.96%	39.76%

Total number of accessions inoculated with each strain is N = 83. Inoculations were repeated at least four times; ten to fifteen plants of each accession were examined.

### Cytological staining and intermediate levels of resistance

**Figure 2:**
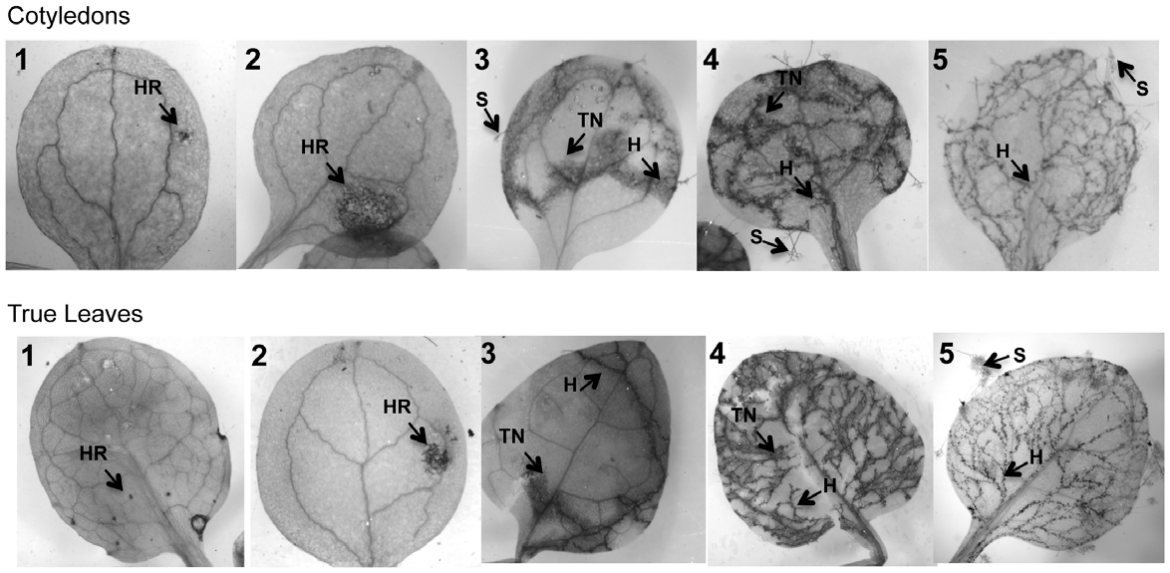
Five phenotypic categories defining race specific interactions between *Hpa* and *Arabidopsis*. Examples of the five phenotypic categories that were observed in cotyledons and true leaves. On cotyledons: 1) *Arabidopsis* Pu2-7 and *Hpa* Maks9, 2) Kz9 and Emco5, 3) Tamm-1 and Emco5, 4) Rmx-A180 and Emoy2, 5) Tsu-1 and Emoy2. On true leaves, 1) Wa-1 and Emoy2, 2) Est1 and Emwa1, 3) Knox-18 and Emoy2, 4) Rmx-A180 and Emoy2, 5) Se-0 and Emco5. Abbreviations: HR – hypersensitive response, H – hyphal growth, TN – trailing necrosis, S – sporangiophores.

Asexual sporulation indicates the ability of *Hpa* to complete its life cycle and propagate, but it does not provide a reliable measurement of the amount of pathogen growth or of the induction of plant immunity. Lactophenol trypan blue staining allows visualization of both intercellular oomycete hyphae as well as the induction of plant cell death [1]. To examine the relationship between host/pathogen interactions on the microscopic level and the pathogen's ability to propagate, we performed lactophenol trypan blue staining of *Arabidopsis* seedlings inoculated with each of the five *Hpa* strains (**Datasets S1, S2, S3, S4, S5**). Based on our observations, all *Arabidopsis/Hpa* interactions could be grouped into five cytological phenotypes, common to cotyledons and true leaves (**Figure 2a** **, Table S1**). We have ranked these plant tissue phenotypes 1 through 5, ranging from resistant and less damaging to fully susceptible and more damaging. The phenotypes are different from each other by two parameters: 1) the extent of pathogen growth and 2) the extent of plant cell death, which can either be radial, forming large circular patches of dying tissue (type 2 phenotype), or linear, tracing the pathogen hyphae (type 3 and 4) (**Figure 2a**). We found a clear correlation between these microscopic phenotypes and the ability of *Hpa* to sporulate (**Table 2** **, Figure S1**). The Type 1 and Type 2 interactions successfully arrested *Hpa* growth and did not support sexual or asexual sporulation. The type 3 phenotype, which showed intermediate levels of pathogen growth and some cell death, supported sporulation in 55% percent of genotype-by-genotype interactions. The type 4 interactions, marked by extensive pathogen growth coupled with plant cell death (commonly referred to as “trailing necrosis”), supported sporulation in 80% of cases. Finally, the type 5 phenotype, which lacks any signs of cell death was correlated with *Hpa* sporulation 100% of the time. This data clearly shows that the ability of *Hpa* to reproduce is linked to its successful colonization of plant tissues, since it increases from phenotype 1 to 5. The cotyledons and true leaves within the same interaction category did not differ in probability of pathogen sporulation (**Figure S1**). About half of the examined interactions were on opposite sides of the phenotypic spectrum (types 1 and 5). The intermediate resistance, manifested by phenotypes 3 and 4 was also prevalent, accounting for 20% to 40% of interactions on cotyledons and 17% to 25% on true leaves (**Table 3**). Interestingly, cotyledons were more prone to expansive plant cell death compared to true leaves, represented by phenotypes 2 and 4 (**Table 3**).

**Table 2:** Association between sporulation and amount of pathogen growth.

Phenotype	1	2	3	4	5
Sporulation on Cotyledons	0.00%	2.86%	54.29%	74.29%	100.00%
Sporulation on True Leaves	0.00%	0.00%	48.00%	85.71%	100.00%

**Table 3:** Percentage of *Arabidopsis* accessions showing interaction phenotypes 1 to 5 on cotyledons and true leaves.

Cotyledons	1	2	3	4	5	N
Emoy2	19.75%	24.69%	17.28%	3.70%	34.57%	81
Maks9	12.82%	20.51%	12.82%	10.26%	43.59%	78
Emco5	32.93%	17.07%	14.63%	6.10%	29.27%	82
Cala2	26.03%	15.07%	16.44%	10.96%	31.51%	73
Emwa1	15.85%	10.98%	26.83%	13.41%	32.93%	82

True Leaves	1	2	3	4	5	N
Emoy2	41.67%	4.17%	16.67%	1.39%	36.11%	72
Maks9	33.82%	5.88%	5.88%	14.71%	39.71%	68
Emco5	55.71%	5.71%	14.29%	2.86%	21.43%	70
Cala2	44.44%	2.78%	13.89%	12.50%	26.39%	72
Emwa1	43.21%	4.94%	17.28%	7.41%	27.16%	81

Interaction phenotypes were scored based on lactophenol trypan blue staining of infected tissue. N - total number of accessions examined. Five to ten plants from each accession were stained and examined. The pictures used to score interactions are provided as supplemental datasets (Datasets S1, S2, S3, S4, S5).

### The developmental effect in *Arabidopsis* disease resistance to *Hpa* is prevalent and race-specific

**Figure 3:**
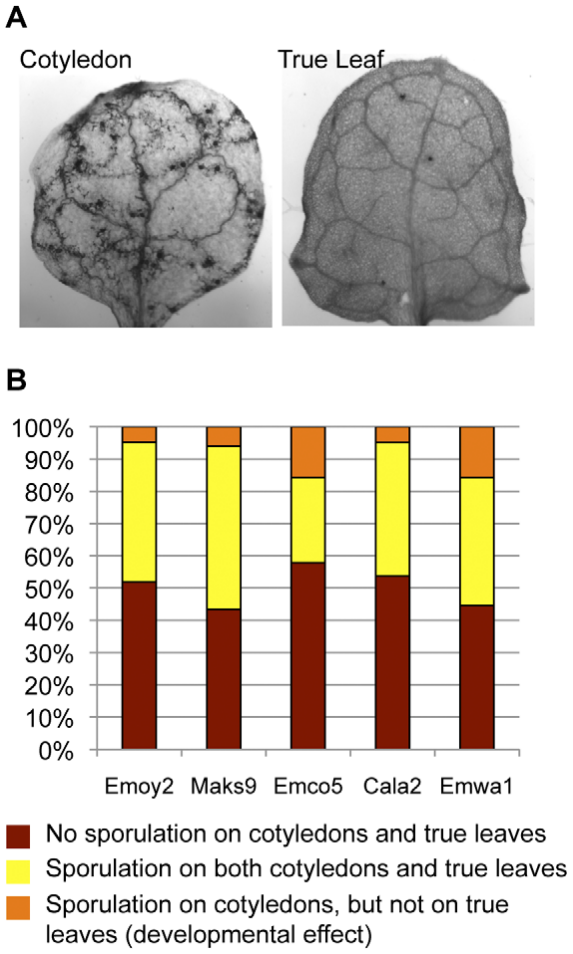
Arabidopsis cotyledons are more susceptible to *Hpa* than true leaves. (A) An example of *Arabidopsis* resistance to *Hpa* showing developmental regulation: CIBC-5 and Emwa1. (B) Prevalence of developmentally controlled resistance among the *Arabidopsis* accessions based on the pathogen's ability to complete its life cycle. Number of accessions sampled, N = 83.

Scoring the cotyledons and true leaves separately allowed us to quantify the prevalence of developmental resistance in the *Arabidopsis*/*Hpa* interactions. We observed that in 20% to 45% of all interactions true leaves exhibited a different phenotype than cotyledons, and in 99% of these cases the extent of pathogen growth was higher on cotyledons than on true leaves (**Figure 3a** **, Table S1**). In a subset of cases, this affected the pathogen's ability to propagate. We observed that in a substantial fraction of accessions, ranging from 4% to 12% depending on the *Hpa* strain applied, cotyledons were consistently more prone to permit pathogen sporulation than true leaves (**Figure 3b**). This did not correlate with overall genealogy of *Arabidopsis* accessions, nor with any particular *Hpa* strain, suggesting that it is race-specific and is regulated by both plant and pathogen factors (**Figure 1**). Since *Hpa* has an equal chance to produce spores on cotyledons and true leaves within the same type of microscopic interactions (**Figure S1**), the resulting difference in sporulation is probably due to quantitative restriction of pathogen growth, and not to suppression of sporulation itself.

### Occurrence of ATR1 and ATR13 effector recognition among *Arabidopsis* accessions

**Figure 4:**
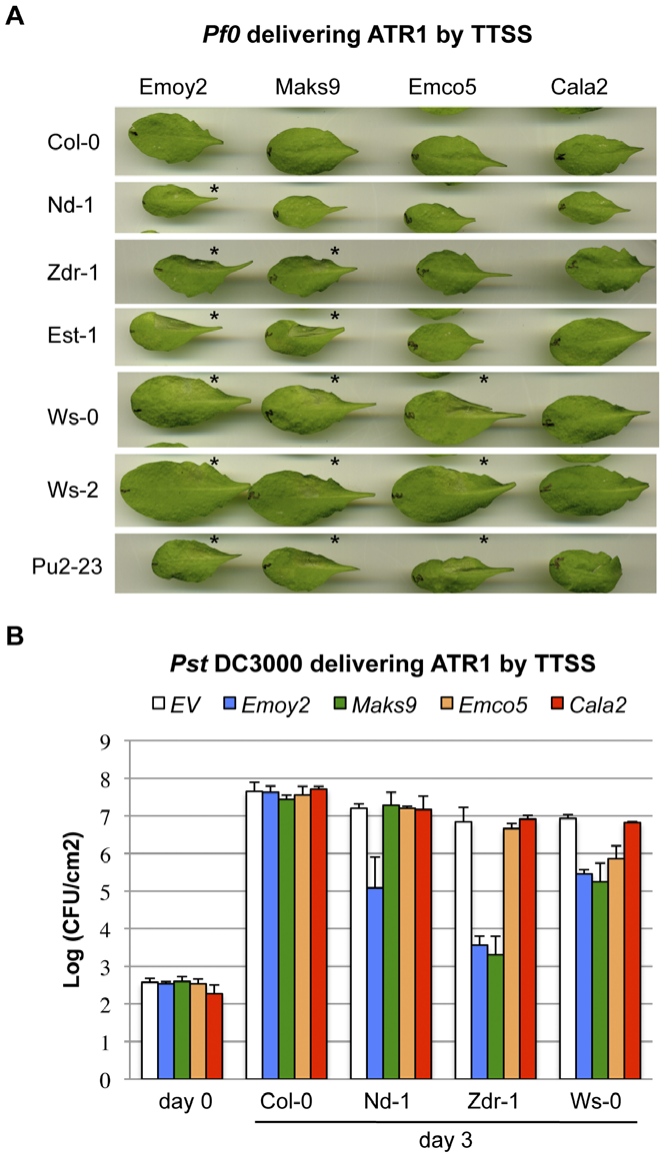
Six *Arabidopsis* accessions recognize the ATR1 effector. *(A)* Recognition of ATR1 delivered by *P. fluorescens* (*Pf*0) TTSS induces HR in six *Arabidopsis* accessions. *Pf*0 delivering pEDV3 ATR1-Emoy2, ATR1-Maks9, ATR1-Emco5 or ATR1-Cala2 was infiltrated in *Arabidopsis* leaf-halves and scored for HR two days post inoculation. The empty vector control (EV) was inoculated on each leaf (bottom left) alongside with ATR1 (top right). Pictures were taken at 24 hours post infiltration. Robust HR responses are denoted with an asterisk. *(B)* Representative growth curves show induction of ATR1-dependent resistance manifested by inhibition of bacterial growth. The same accessions as above were hand-infiltrated with *P. syringae* pv. tomato (*Pst*) DC3000 delivering pEDV3 EV, ATR1-Emoy2, ATR1-Maks9, ATR1-Emco5 or ATR1-Cala2 and bacterial titers determined at 0 and 3 days post infection. The growth curves shown illustrate four different recognition specificities of ATR1 alleles. Additional growth curves are shown in Figure S2.

**Figure 5:**
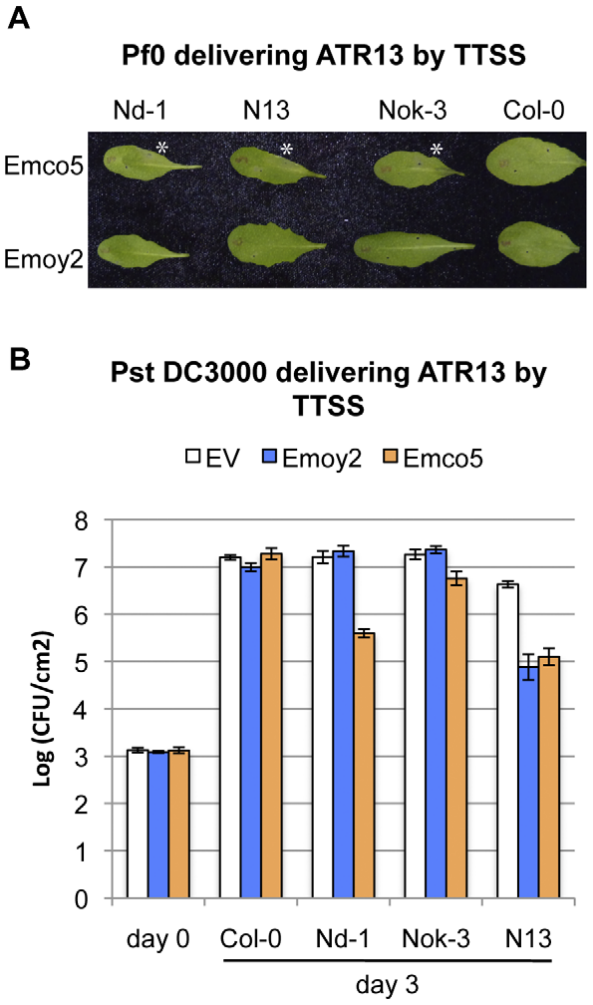
Three *Arabidopsis* accessions recognize the ATR13 effector. *(A)* HR assay of ATR13-Emco5 and Emoy2 delivered by *P. fluorescens* (*Pf*0) TTSS, 24 hours post infiltration. Robust HR responses are denoted with an asterisk. *(B)* Growth assay of *Pst* DC3000 delivering ATR13 variants on Col-0, Nd-1, N13 and Noks-3 accessions from the Nordborg collection.

*Hpa*, being an obligate biotrophic pathogen, is not easily genetically manipulated. Therefore, in order to assay contribution of individual effectors to overall resistance, we utilized a previously developed bacterial Type III Secretion System (TTSS) delivery strategy [19], [20]. We adopted non-pathogenic *Pseudomonas fluorescens* (*Pf0*) supplemented with the TTSS machinery to minimize contribution of endogenous bacterial Type III effectors present in other strains of pathogenic *Pseudomonas* [26]. This system allowed us to rapidly score for recognition of ATR1 and ATR13, visualized as macroscopic cell death, in a number of *Arabidopsis* accessions with minimal background. Delivery of ATR1 and ATR13 protein by *Pf0* into accessions known to contain the cognate *R*-genes, *RPP1* (Nd-1 and Ws-0) and *RPP13* (Nd-1) induced a strong effector-dependent hypersensitive reaction (HR) at about 24 to 48 hours post inoculation (**Figure 4a**). Using HR as our initial assay, we screened *Arabidopsis* accessions with four polymorphic alleles of *ATR1*: *Emoy2* (gi61660946), *Maks9* (gi61660952), *Emco5* (gi61660954), and *Cala2* (gi61660958), and two alleles of *ATR13*: *Emoy2* (gi58042853) and *Emco5* (gi58042859). We found four additional accessions that were able to recognize ATR1 (**Figure 4** **, Figure S2**). Two of the accessions, Ws-2 and Pu2-23, had the same recognition specificity as Ws-0, and were able to recognize ATR1-Emoy2, Maks9 and Emco5, but not Cala2. Another two accessions, Zdr-1 and Est-1, showed altered recognition specificity, and recognized ATR1-Emoy2 and Maks9, but not ATR1-Emco5 or Cala2. The only accession specifically recognizing ATR1-Emoy2 and not any other allele tested was Nd-1. To further validate our findings, we performed bacterial growth curve assays delivering ATR1 by TTSS of *Pseudomonas syringae* pv. tomato (*Pst*) DC3000 (**Figure 4b** **, Figure S2**). We observed perfect agreement between the HR induced in response to ATR1 delivered by *Pf0* (**Figure 4a**) and restriction of *Pst* DC3000 growth (**Figure 4b** **, Figure S2**). Unlike Sohn *et al.*, we did not observe enhanced bacterial virulence in the presence of ATR1 (**Figure 4b** **, Figure S2**). The occurrence of ATR13 recognition in *Arabidopsis* accessions outside of the United Kingdom proved to be even more rare. Only two accessions, Noks-1 and N13, in addition to the previously known Nd-1 were capable of eliciting ATR13-Emco5 dependent resistance (**Figure 5**).

**Figure 6:**
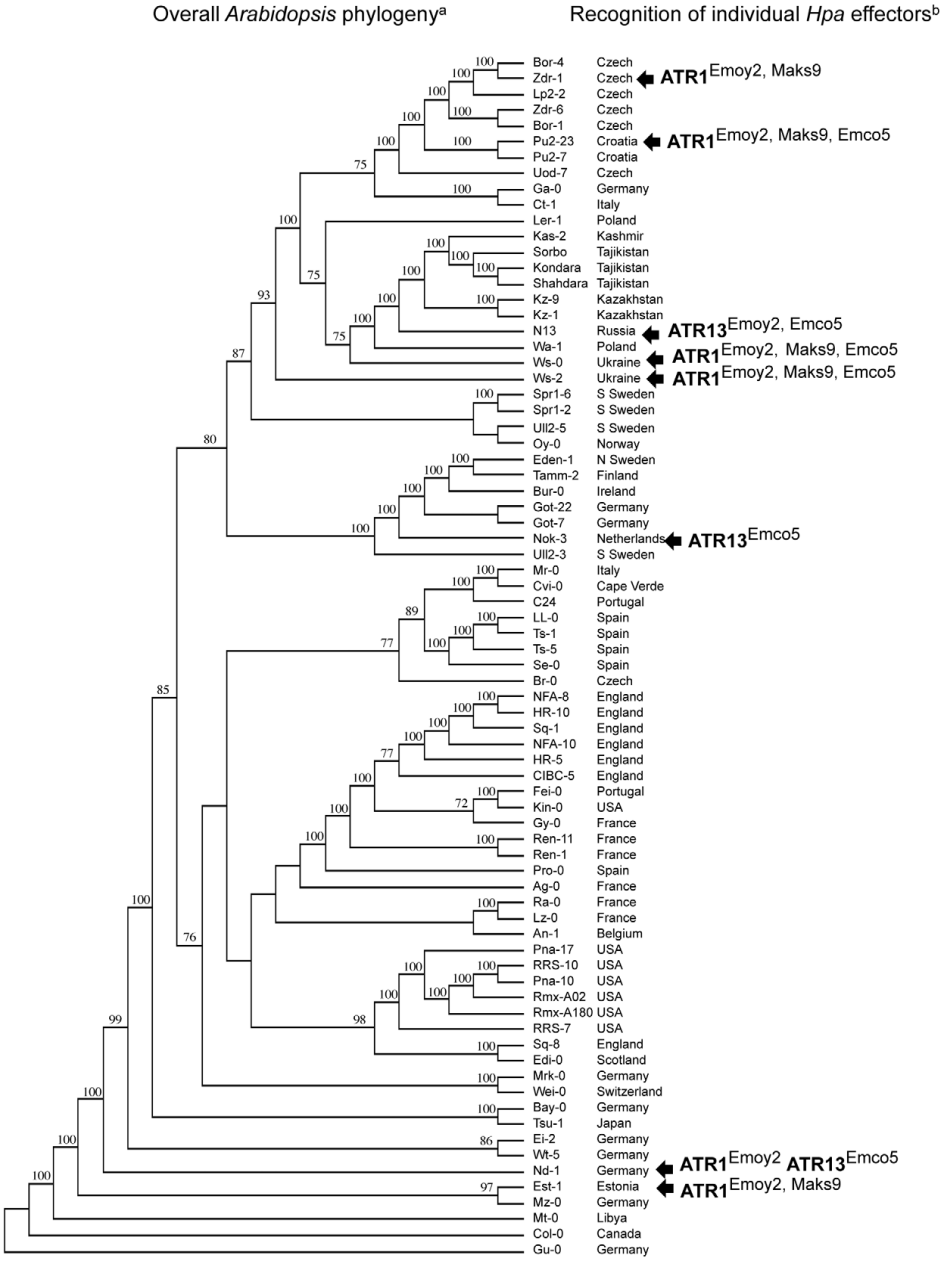
Phylogenetic relationship of accessions that recognize ATR1 effector relative to overall *Arabidopsis* phylogeny. The phylogenetic tree on the left represents the genome-wide relationship between accessions based on small nucleotide polymorphism data as in Figure 1 [13]. *Arabidopsis* accessions capable of recognizing subsets of ATR1 and ATR13 alleles are marked by arrows with the corresponding recognition specificities.

Finally, we compared the evolution of ATR1 and ATR13 recognition with overall *Arabidopsis* genealogy (**Figure 6**). The *Arabidopsis* accessions capable of recognizing ATR1 or ATR13 did not form a single evolutionary clade (**Figure 6**). Moreover, several accessions with the same recognition range were more distantly related to each other than to those with altered recognition specificities (**Figure 6**). These results showed that being the closest relatives with respect to overall genomes had little predictive power over the ability to recognize a specific oomycete effector.

### *Hpa* strain Emco5 escapes recognition

**Figure 7:**
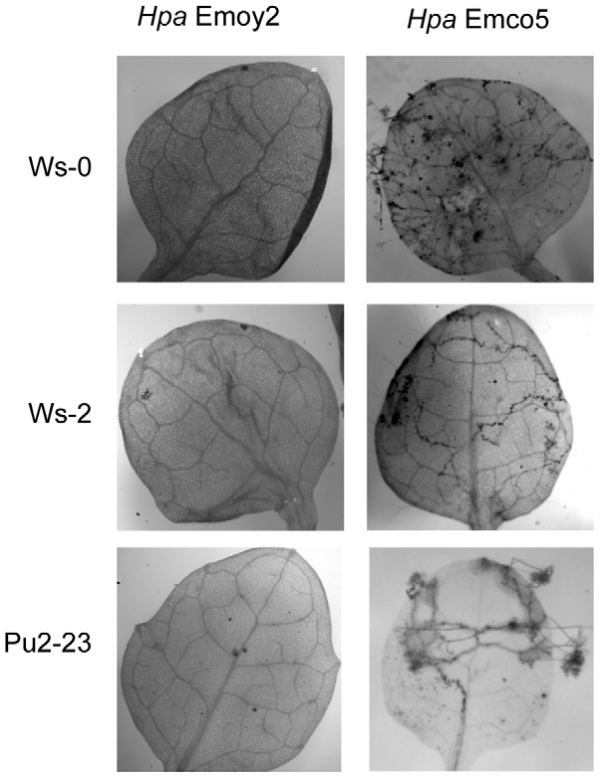
*Hpa* Emco5 escapes recognition. The accessions Ws-0, Ws-2 and Pu2-23 are able to induce defense responses to *Hpa* Emoy2, but not *Hpa* Emco5. Seedlings were stained with lactophenol trypan blue 7 days post-infection, true leaves are depicted.

The possibility to examine individual oomycete effectors allowed us to evaluate their relative contributions to overall disease resistance among *Arabidopsis* accessions. The contribution of ATR1 towards resistance varied depending on individual *Hpa* strain (**Table 4**). Interestingly, recognition of ATR1-Emco5 did not protect plants against *Hpa* Emco5 infection (**Table 4** **,** **Figure 7**). The ability to recognize ATR1-Emco5 was not limited to the bacterial delivery system since specific recognition of ATR1-Emco5 by Arabidopsis Ws-0 was also observed in a biolistic bombardment assay [23] and by *Agrobacterium*-mediated transient expression [22]. The ATR1-Emco5 transcript has been shown to be present in the pathogen [23], eliminating the possibility that this discrepancy is due to lack of gene expression. Since *Hpa* is normally propagated at 18°C in high humidity and bacterial assays are conducted at room temperature (around 24°C), we addressed whether the discrepancy could be due to differences in growth conditions. We found no evidence for temperature or humidity regulation of ATR1 recognition, as the *Arabidopsis* plants were able to induce HR at 18°C with the same timing and intensity as at 20°C or 24°C. From this data we hypothesize that the *Hpa* Emco5 pathogen has acquired the ability to prevent/suppress recognition of ATR1.

**Table 4:** Comparison between *Arabidopsis* response to *Hpa* strains and to individual alleles of ATR1 effector delivered by TTSS.

Response category		Number of accessions responding to *Hpa* strain/ATR1 allele				Explanation
*Hpa* a	ATR1^b^	Emoy2	Maks9	Emco5	Cala2	
Susceptible	No response	40	47	32	38	No resistance.
Resistant	No response	37	31	48	44	Resistance is specified by other RPP/ATR interactions.
Resistant	Recognized	6	5	0	0	Resistance is specified in part by ATR1/RPP1.
Susceptible	Recognized	0	0	3	0	RPP1 is functional, yet resistance is actively suppressed.

a inoculation with the whole pathogen,

b delivery of ATR1 by Type III Secretion System (TTSS).

## Discussion

In this study, we phenotypically characterized approximately 400 *Arabidopsis/Hpa* interactions and analyzed these interactions from several different angles. Although some of the phenotypes we describe have been noted before, conducting a large-scale study allowed us to differentiate the “rules” from the “exceptions” in *Arabidopsis*/*Hpa* interactions. We postulate the following principles: i) there is a prevalence of developmental control of *Arabidopsis* immunity, ii) there are several prevalent levels of intermediate resistance, iii) a relatively small percentage of resistance is attributable to recognition of individual *Hpa* effectors, such as ATR1 or ATR13, iv) recognition of oomycete effectors in *Arabidopsis* is evolutionary dynamic and does not correlate with overall genomic relatedness, and v) pathogen is able to escape recognition despite functional *ATR/RPP* interactions.

### Intermediate resistance plays a major role in *Arabidopsis/Hpa* interactions

We observed that intermediate resistance is prevalent among *Arabidopsis/Hpa* interactions. We could distinguish two factors that conferred intermediate phenotypes: the level of pathogen growth and the difference in host response depending on developmental stage. The intermediate levels of pathogen growth and its corresponding ability to sporulate were often inversely correlated to a plant cell death response trailing the pathogen hyphae. Since this “trailing necrosis” phenotype was associated with reduced sporulation and provided little benefit to the pathogen, it is unlikely to be a disease-related necrosis. More likely, it represents a form of hypersensitive response, which is unable to completely control pathogen growth due to partially compromised plant immunity. For example, this trailing necrosis phenotype has also been observed in *Arabidopsis* mutants impaired in basal defense, such as *pad4* or *ndr1* [27].

Previously, our knowledge about developmental effects in *Arabidopsis* disease resistance to *Hpa* was limited to one isolated case of *Hpa* Emco5 interacting with a common lab accession of *Arabidopsis* Col-0 [18]. Our results show that developmental variation in resistance to *Hpa* is prevalent among *Arabidopsis* populations worldwide. Additionally, it is evident that the discrepancy in responses between cotyledons and true leaves depends on both the genotype of the plant and the genotype of the pathogen. The effect is always directional with the more juvenile organs, cotyledons, being more susceptible to the pathogen than true leaves. This effect is largely due to enhanced ability of the pathogen to colonize cotyledons and establish intercellular growth. The factors controlling this phenotypic difference between different plant organs are still unknown. Since the 30 known *RPP* genes were identified based on functional resistance against *Hpa* in cotyledons [3], we still do not know the primary source for the differential adult resistance in true leaves. Recently, computational genome-wide association analyses predicted that only 6 loci would specify the majority of *Arabidopsis* resistance to *Hpa* in true leaves [17], but this remains to be validated. Since our data shows that developmentally controlled immunity follows race-specific interactions, it is unlikely that it is determined by a single gene exerting global control on resistance pathways. One explanation is that a subset of currently unidentified *R* genes is under developmental control and is only functional in true leaves. An alternative explanation can be postulated from the pathogen's perspective. In a subset of interactions, *Hpa* could be actively suppressing some of the resistance pathways in cotyledons. The latter hypothesis is supported by a recent study showing that many pathogen-derived effectors share a set of common targets [28], some of which could be tissue-specific. Both hypotheses imply that there is a difference in the disease resistance mechanisms in cotyledons and true leaves. A variety of plant phenotypes linked to phase change have recently been investigated and were shown to be controlled by small RNA molecules [29]. It would be important to investigate whether small RNAs also have a role in developmental regulation of plant immunity. Our data can be used to dissect the developmental effects through genetic crosses. Complemented with advanced sequencing technologies, it should be possible to map the source of developmental resistance in a variety of accessions.

### Recognition of individual effector variants is rare among *Arabidopsis* accessions

Pathogen-derived effector molecules, which serve as molecular triggers of plant defenses, form a class of extremely diverse and fast-evolving proteins. These effectors alongside with plant R proteins are molecular factors that specify dynamics of host/pathogen interactions on the evolutionary scale. Following individual effector/*R* gene interactions, we can observe their contribution to the ultimate outcome of disease or resistance in a natural pathosystem. Several previous studies in *Arabidopsis* examined contribution of individual bacterial Type III secretion system effectors. It has been shown that recognition of conserved bacterial effectors is widespread among *Arabidopsis* accessions and correlates well with the overall genomic variation between different *Arabidopsis* accessions [30], supporting a relatively slow rate of evolution of the cognate *R* genes. On the other hand, oomycete effectors and corresponding *R* genes show signatures of rapid evolution [8], [11], [16], [23], suggesting different interaction dynamics on a population level. We looked at the prevalence of ATR1 and ATR13 effector recognition among *Arabidopsis* accessions and found six accessions that recognized different subsets of ATR1 variants and three that recognized ATR13. Compared to what has been observed for bacterial effectors [30], the distribution of ATR1 and ATR13 recognition is extremely rare. Furthermore, these accessions do not form a single cluster in the *Arabidopsis* phylogeny, suggesting that recognition of ATR1 and ATR13 could have evolved independently in different lineages. A similar conclusion was proposed in previous analyses of ATR13 recognition [24]. In the case of ATR13, it has been shown that its recognition can be specified by independent loci. Additionally, the same locus that specifies ATR13 recognition in some accessions can recognize a different effector in others [24]. This shows that *R* genes specifying resistance against highly divergent oomycete effectors do not necessarily form families based on the effector they recognize. This type of disease resistance, targeted at monitoring rapidly evolving molecules, is different from more slowly evolving *Arabidopsis* R genes, such as *RPM1*, *RPS2* or *RPS4*, that recognize effectors based on their enzymatic activity; the latter class of effectors is normally found under balancing selection [31]. The rapid evolution of recognition of oomycete effectors challenges the gene-for-gene model of plant immunity. Indeed, if genes arising from a single locus possess the potential to recognize unrelated effectors, and genes arising from multiple loci have acquired the ability to recognize the same effector, we might need to update our nomenclature. Currently, the *RPP1* locus contains three functional genes that are capable of recognizing unrelated ATR effectors [4]. Similarly, *RPP4* and *RPP5*, which recognize ATR4 and ATR5, respectively, are located at the same locus in different *Arabidopsis* accessions [7]. One of the most diversified *R* genes known today, *RPP13*, has been shown to recognize at least two different effectors [8], [24]; similarly, the ATR13 effector can be recognized by different loci [24]. This breaks the cognate relationship usually attributed to effectors and *R* genes. Instead, it seems that a highly adaptive pool of *R* genes provides genetic potential for maintaining effector recognition or establishing new recognition specificities.

### *Hpa*'s escape from recognition

The *Arabidopsis/Hpa* interactions have yet another level of complexity: the ability of the pathogen to escape host recognition without major modifications of effector gene sequence. The recognition of the ATR1-Emco5 allele by RPP1-WsB has been previously demonstrated both in *Arabidopsis* [23] and by transient *Agrobacterium*-mediated expression in *Nicotiana tabacum* [22]. However, all of the accessions that are able to specifically recognize ATR1-Emco5 when delivered by *Pseudomonas* are susceptible to the *Hpa* Emco5 strain. This discrepancy cannot be attributed to genetic modifications of *ATR1* and *RPP1* coding sequences. There are several alternative explanations for this pathogen's escape from recognition. First, although it has been shown that *ATR1-Emco5* is expressed in *Hpa*, we cannot exclude the possibility that the effector protein is not properly translocated into the host where it would be recognized by associating with the LRR of RPP1. An alternative hypothesis is the active suppression of ATR1 recognition or downstream signaling events by ATR1 or another *Hpa* effector. Suppression of effector-triggered immunity has been widely studied in the case of bacterial effectors, but has yet to be demonstrated in *Arabidopsis/Hpa* interactions. The ATR1-Emco5 interaction with RPP1 can serve as a basis for uncovering immunity suppressors among the predicted *Hpa* effectors. Additionally, such suppression can introduce substantial noise to the genotype-based predictions about effector/*R gene* interactions, and should be accounted for in evolutionary analyses.

Our study opens exciting new avenues for investigations of plant/pathogen interactions using the *Arabidopsis*/*Hpa* pathosystem. Our results point at ways to uncover the developmental regulation of plant immunity, provide a clear strategy of expanding the currently narrow pool of known ATR/RPP interactions and suggest active suppression of plant immunity by *Hpa*. Importantly, if non-allelic *R* genes recognize the same effectors, and, on the other hand, allelic *R* genes recognize different effectors, an update to the nomenclature of *R* genes might be necessary to keep track of recognition specificities towards rapidly evolving effectors. Understanding the mechanisms controlling the dynamic equilibrium of host/pathogen interactions based on genetic diversity will allow for development of more sustainable agricultural strategies, which presently rely on genetically restricted plant species.

## Materials and Methods

### Strains and growth conditions

*Escherichia coli* DH5α used for cloning and propagation of pEDV3 constructs was routinely grown at 37°C in Luria Bertani broth media or agar plates supplemented with 10 µg/mL gentamycin. *Pseudomonas* strains were propagated at 28°C. *Pseudomonas fluorescens (Pf0)* was grown on Pseudomonas Agar solid medium supplemented with 50 µg/mL tetracyclin, 30 µg/mL chloramphenicol and 150 µg/mL gentamycin and *Pseudomonas syringae* pv. tomato DC3000 (*Pst* DC3000) was grown on NYGA solid medium supplemented with 100 µg/mL rifampicin and 5 µg/mL gentamycin.

### *Arabidopsis* growth conditions, *Hyaloperonospora arabidopsidis* propagation and inoculations

The Nordborg collection of 95 *Arabidopsis* accessions, a subset of which was used in this study, was described previously [15] and can be obtained from the Arabidopsis Biological Resource Center (ABRC, Ohio State University). A fraction of plants that routinely failed to germinate or had very delayed germination were dropped from the analysis, reducing the number of accessions from the original 95 to 83. For each experiment, a complete set of plants was grown in 2×2 inch pots and maintained at the same conditions (24°C, 8/16 hr light-dark cycle). *Hpa* strains were asexually propagated as described previously [1], and spray-inoculated on two-week-old *Arabidopsis* seedlings with the first set of true leaves. Conidiospore density in the inoculum was ∼10^5^ to 10^6^ spores/mL. After inoculations, plants were transferred to an 18°C chamber with high humidity. Inoculations were repeated at least three to four times. Sporangiophore formation was recorded at 7–8 days post inoculation, when the *Hpa* life cycle had been completed. Lactophenol trypan blue staining was done at 7–8 days post inoculation, following a previously described protocol [1] with minor modifications. Around 5–8 plants of each genotype were collected in 1.5 mL Eppendorf tubes with 0.5 mL of lactophenol trypan blue staining solution. The tubes were boiled for 2 minutes and incubated on the bench from 2 hours to overnight. Seedlings were subsequently transferred to 96 well plates and de-stained in 0.2 mL of chloral hydrate overnight.

### Type III effector delivery, hypersensitive response assays and *Pseudomonas* growth curves

The ATR1Δ49-Emoy2 and Cala2, as well as ATR13-Emoy2 and Emco5 alleles cloned into the Type III delivery vector pEDV3 were kindly provided by Jonathan Jones (Sainsbury Labs, United Kingdom)[20]. The Maks9 and Emco5 alleles of ATR1Δ49 were sub-cloned into pEDV3 employing SalI/BamHI restriction enzyme cutting sites in the vector.

All effector constructs as well as empty vector pEDV3 were conjugated into *Pf*0 TTSS [26] and *Pst* DC3000 via triparental mating using the *E. coli* HB101 pRK600 helper strain. For plant inoculations, strains were grown from glycerol stocks on agar plates with appropriate antibiotics for 1–2 days. The hypersensitive response (HR) assays were conducted with *Pf0* inoculated at OD_600 nm_ = 1.0 (10^7^ CFU/mL) into young, fully expanded leaves of 5–6 week old plants. Empty vector pEDV3 was included on each leaf as a negative control to monitor for any background plant response to *Pf0*. The HR was scored at 1–3 days post inoculation. Bacterial growth assays were conducted with *Pst* DC3000 using the syringe hand-inoculation method as described previously [32]. Bacterial titer was determined at 0 and 3 days post inoculation.

### Reconstruction of *Arabidopsis* phylogeny

The 205K small nucleotide polymorphism (SNP) data, published by Atwell *et al.* [13], was obtained from the *Arabidopsis thaliana* polymorphism database (https://cynin.gmi.oeaw.ac.at/home/resources/atpolydb). The SNP data was available for 72 of the 83 accessions used in this study, thus limiting our phylogenetic analysis to those 72 accessions. The phylogenetic relationship was constructed using the Phylip 3.66 software [33]. Specifically, bootstrapping was performed using *seqboot* with 100 replicates, the distance matrices were built using the *dnadist* algorithm with default parameters, the trees were made using the Neighbor-Joining algorithm, and the consensus tree was derived with the *consense* program. The tree was visualized using the TreeView X program [34].

## Supporting Information

Figure S1 **Prevalence of pathogen sporulation associated with individual** ***Hpa/Arabidopsis*** **interaction phenotypes.** Each data point in this analysis presents one *Arabidopsis* accession interacting with one *Hpa* strain. Number of genotype-by-genotype interactions sampled, N = 396 for cotyledons, N = 363 for true leaves.
(PDF)

Figure S2 **Bacterial growth assays on Pu2-23, Est-1 and Ws-2.** Additional bacterial growth assays showing recognition of different ATR1 alleles by *Arabidopsis* accessions (A) Pu2-23, (B) Est-1 and (C) Ws-2.
(PDF)

Table S1 **Phenotypic responses of 83** ***Arabidopsis*** **accessions to** ***H. arabidopsidis*** **strains Emoy2, Maks9, Emco5, Cala2 and Emwa1.** Accessions are listed in alphabetical order. Coloring scheme: brown – absence of asexual sporulation on both cotyledons and true leaves, orange – sporulation is present on cotyledons, but not on true leaves, yellow – sporulation is present on both cotyledons and true leaves. Numbers indicate phenotypic scoring (type 1 to 5, described in the text) for the interactions that have been analyzed by microscopy (see Datasets S1, S2, S3, S4, S5), n – data not available. The first number in each column corresponds to the score on cotyledons, the second number to the score on true leaves.
(XLS)

Dataset S1 **Images of the trypan blue-stained** ***Arabidopsis*** **cotyledons and true leaves inoculated with** ***Hpa*** **Emoy2.**
(PDF)

Dataset S2 **Images of the trypan blue-stained** ***Arabidopsis*** **cotyledons and true leaves inoculated with** ***Hpa*** **Maks9.**
(PDF)

Dataset S3 **Images of the trypan blue-stained** ***Arabidopsis*** **cotyledons and true leaves inoculated with** ***Hpa*** **Emco5.**
(PDF)

Dataset S4 **Images of the trypan blue-stained** ***Arabidopsis*** **cotyledons and true leaves inoculated with** ***Hpa*** **Cala2.**
(PDF)

Dataset S5 **Images of the trypan blue-stained** ***Arabidopsis*** **cotyledons and true leaves inoculated with** ***Hpa*** **Emwa1.**
(PDF)
