# Supplementary figures and images for: Global Analysis of Arabidopsis/Downy Mildew Interactions Reveals Prevalence of Incomplete Resistance and Rapid Evolution of Pathogen Recognition
Source: PLoS One. 2011 Dec 14;6(12):e28765. doi: 10.1371/journal.pone.0028765 (PMC3237489; doi:10.1371/journal.pone.0028765)

Supplemental Figure 1.

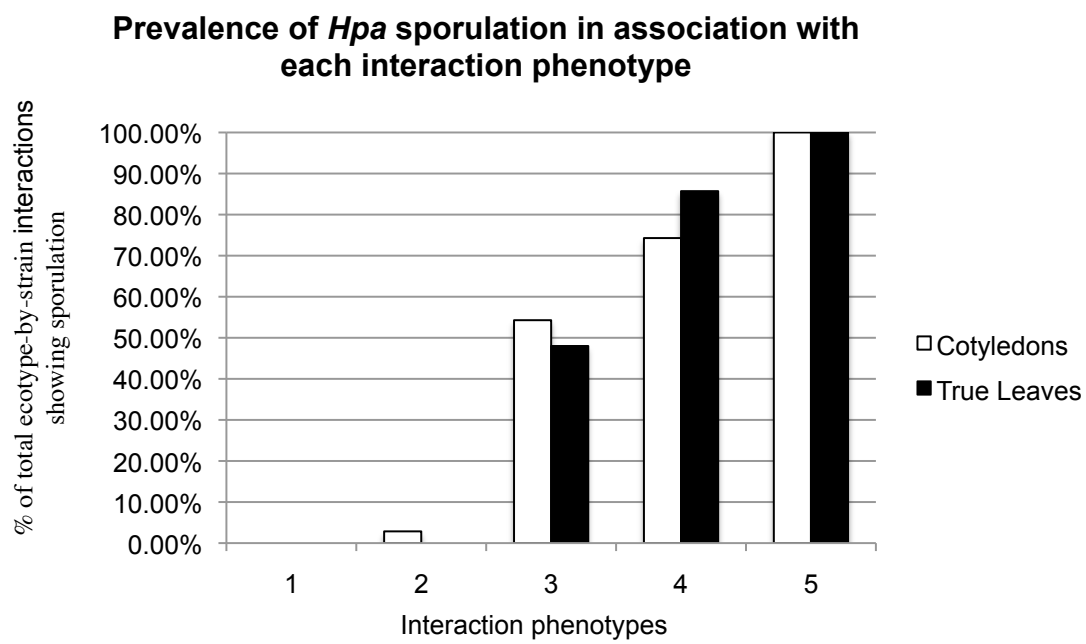

Supplement: Figure S1 — Prevalence of pathogen sporulation associated with individual Hpa/Arabidopsis interaction phenotypes. Each data point in this analysis presents one Arabidopsis accession interacting with one Hpa strain. Number of genotype-by-genotype interactions sampled, N = 396 for cotyledons, N = 363 for true leaves. (PDF) [file pone.0028765.s001.pdf]

Supplemental Figure 2.

Pst DC3000 delivering ATR1 by TTSS

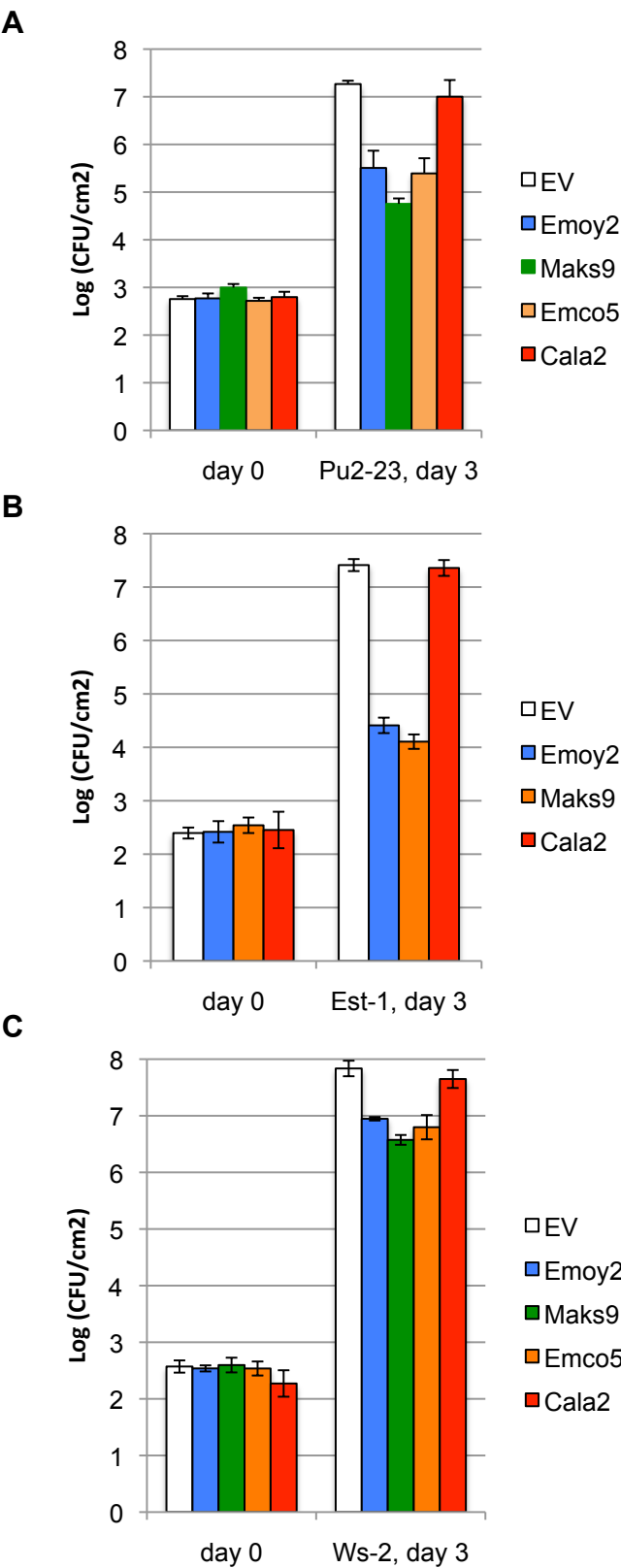

Supplement: Figure S2 — Bacterial growth assays on Pu2-23, Est-1 and Ws-2. Additional bacterial growth assays showing recognition of different ATR1 alleles by Arabidopsis accessions (A) Pu2-23, (B) Est-1 and (C) Ws-2. (PDF) [file pone.0028765.s002.pdf]
