# Supplementary material for: Global Analysis of Arabidopsis/Downy Mildew Interactions Reveals Prevalence of Incomplete Resistance and Rapid Evolution of Pathogen Recognition
Source: PLoS One. 2011 Dec 14;6(12):e28765. doi: 10.1371/journal.pone.0028765 (PMC3237489; doi:10.1371/journal.pone.0028765)

Supplemental Dataset 1. *Hpa* Emoy2

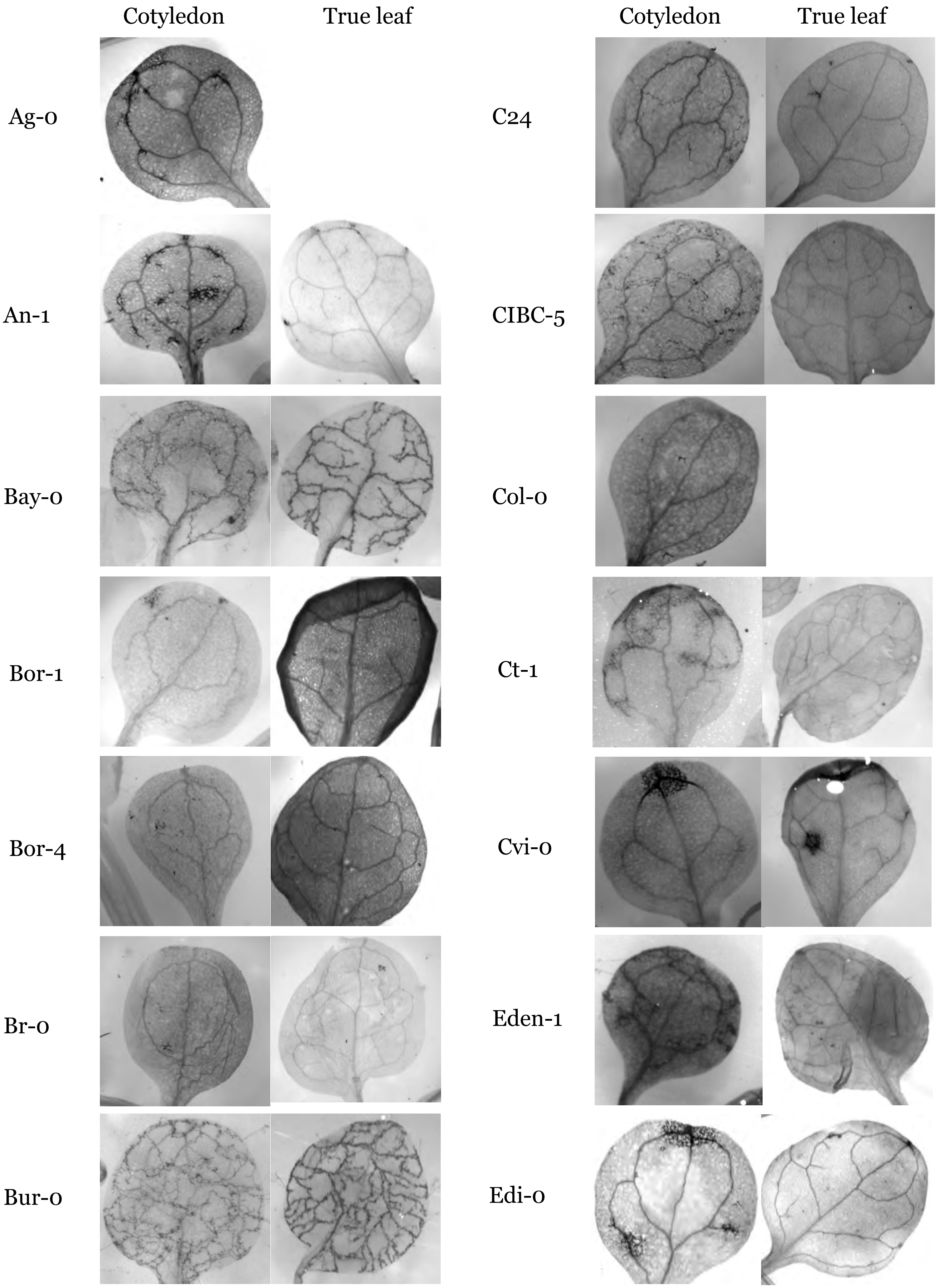

Supplemental Dataset 1. *Hpa* Emoy2

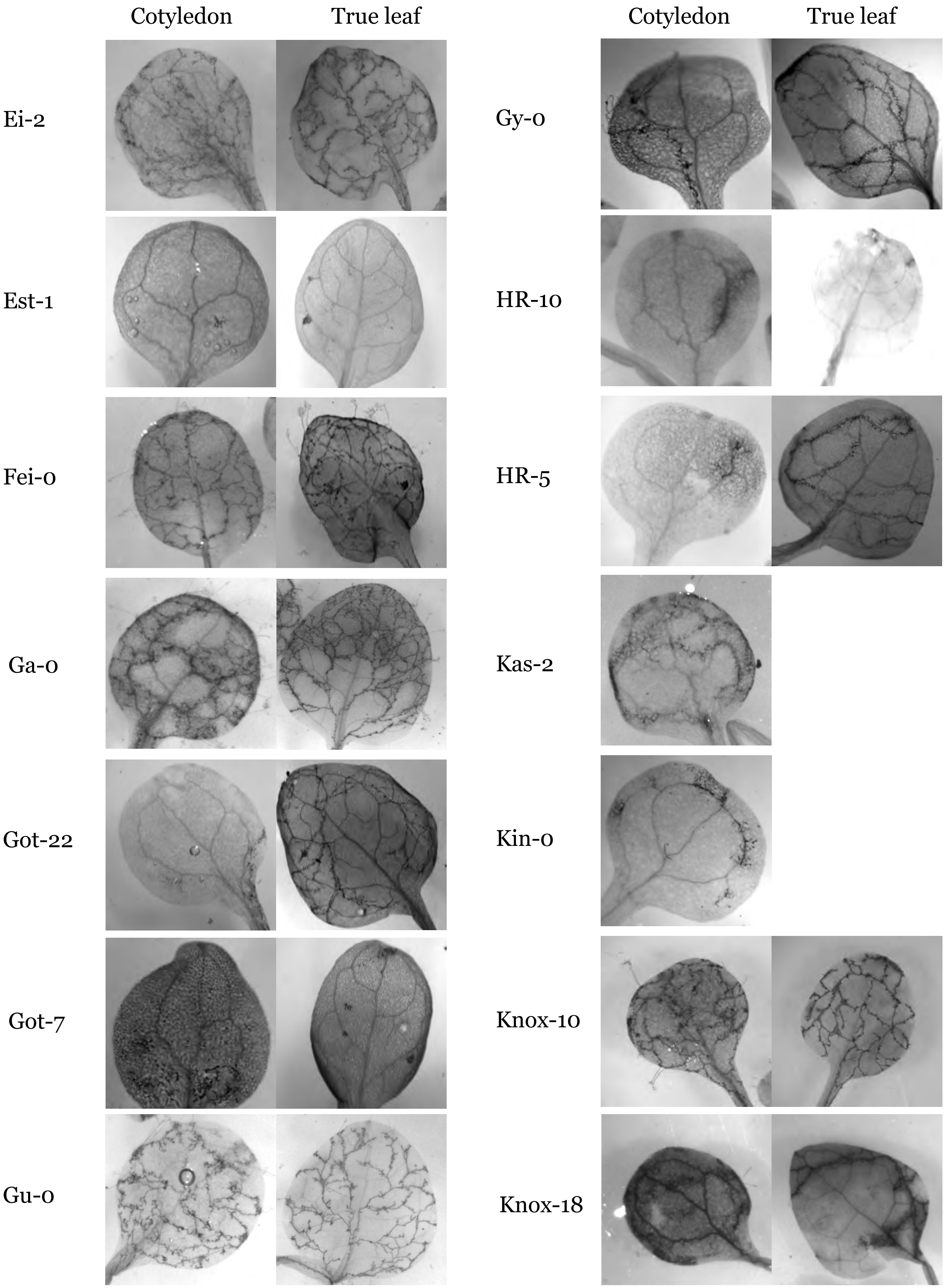

Supplemental Dataset 1. *Hpa* Emoy2

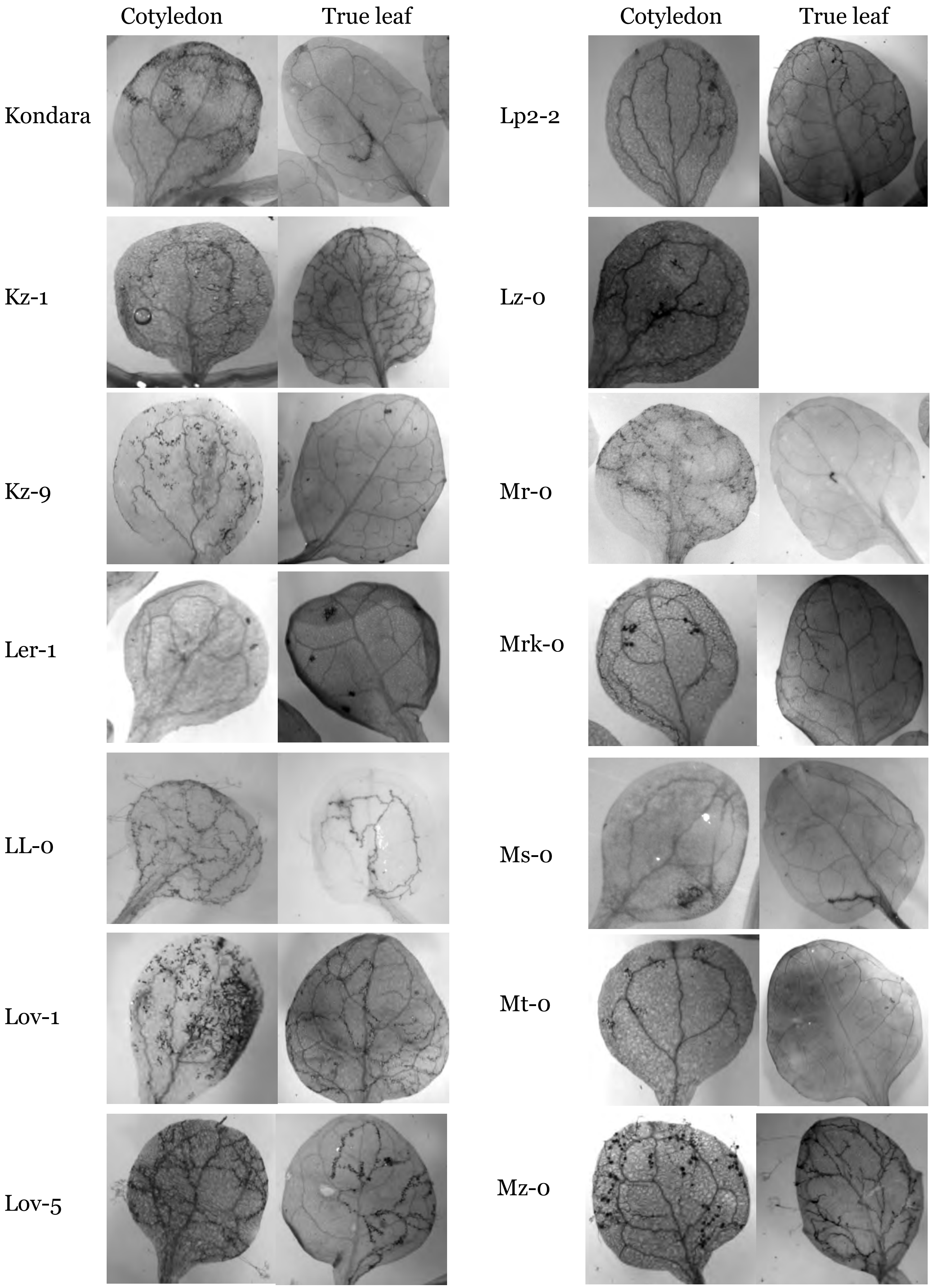

Supplemental Dataset 1. *Hpa* Emoy2

Cotyledon | True leaf

N13

Nd-1

NFA-8

NFA-10

Nok-3

Omo2-3

Oy-0

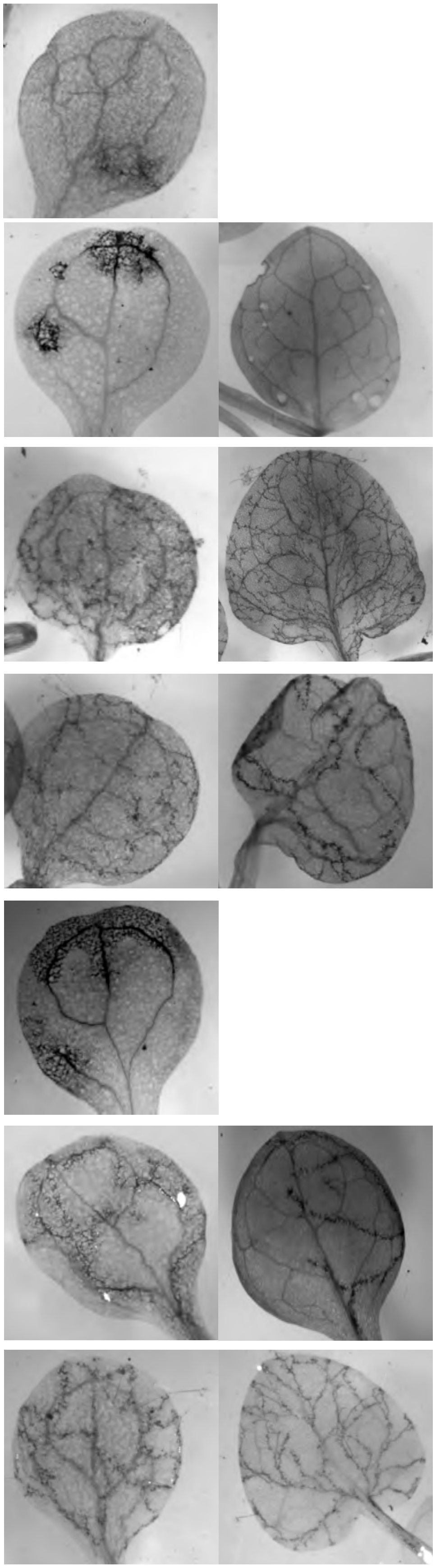

Cotyledon | True leaf

Pna-17

Pna-10

Pro-0

Pu2-23

Pu2-7

Ra-0

Ren-1

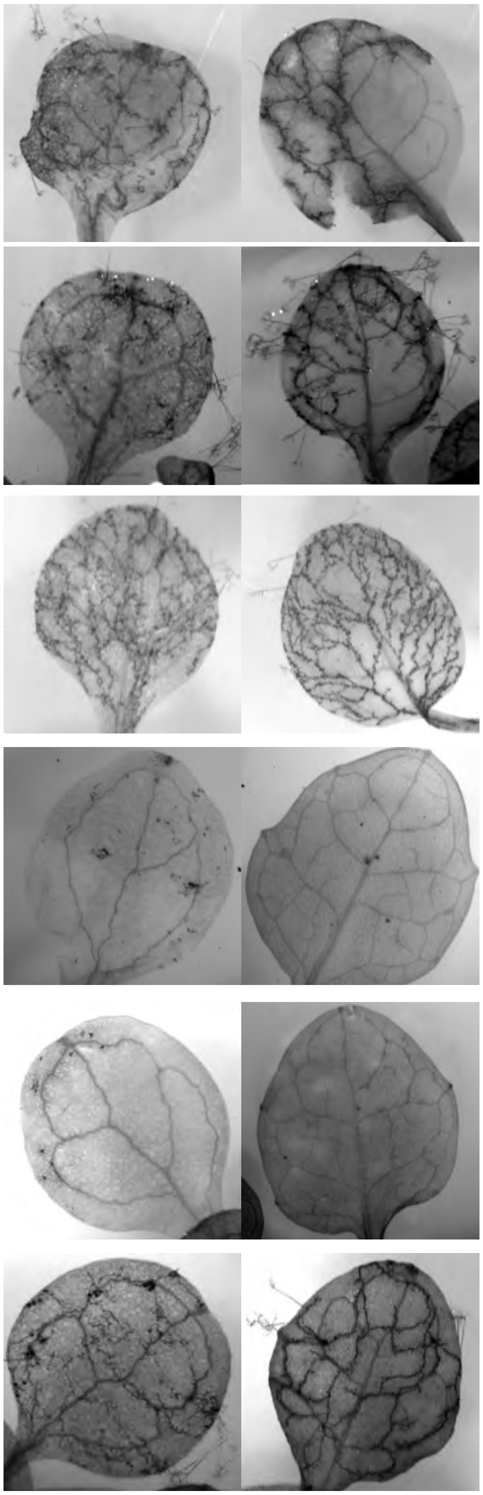

Supplemental Dataset 1. *Hpa* Emoy2

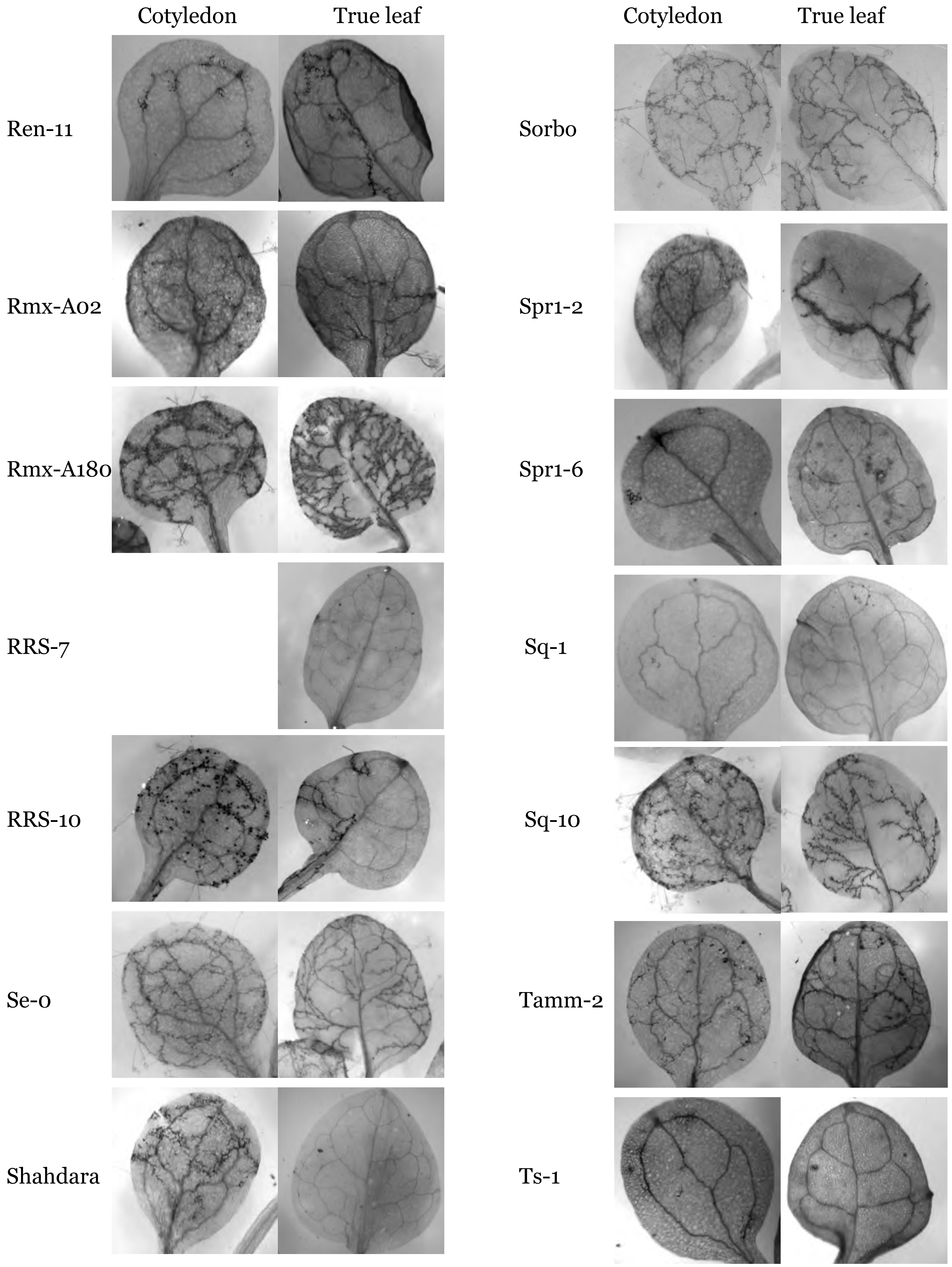

Supplemental Dataset 1. *Hpa* Emoy2

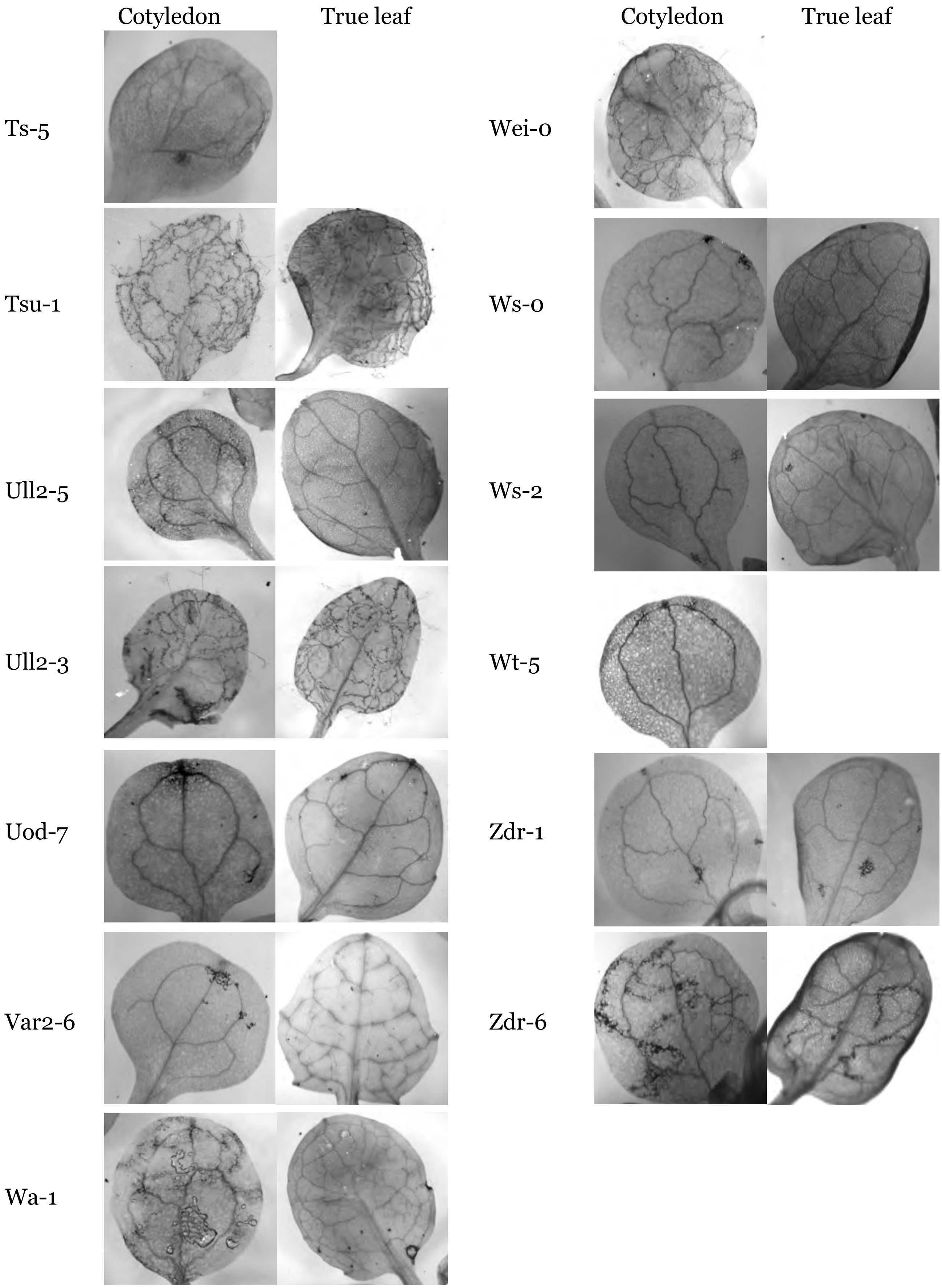

Supplement: Dataset S1 — Images of the trypan blue-stained Arabidopsis cotyledons and true leaves inoculated with Hpa Emoy2. (PDF) [file pone.0028765.s004.pdf]
