# Supplementary material for: Global Analysis of Arabidopsis/Downy Mildew Interactions Reveals Prevalence of Incomplete Resistance and Rapid Evolution of Pathogen Recognition
Source: PLoS One. 2011 Dec 14;6(12):e28765. doi: 10.1371/journal.pone.0028765 (PMC3237489; doi:10.1371/journal.pone.0028765)

Supplemental Dataset 2. *Hpa* Maks9

Cotyledon | True leaf

Ag-0

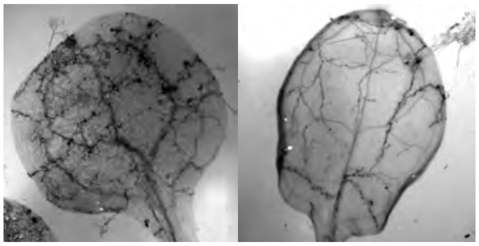

An-1

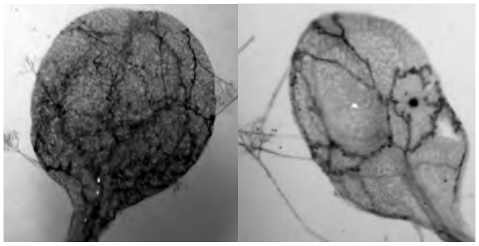

Bay-0

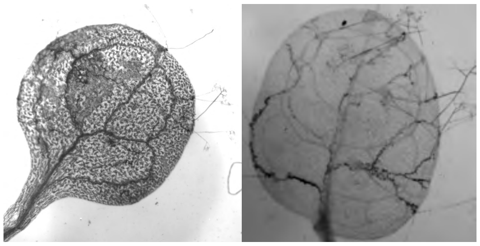

Bor-1

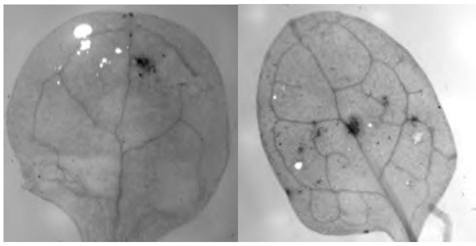

Bor-4

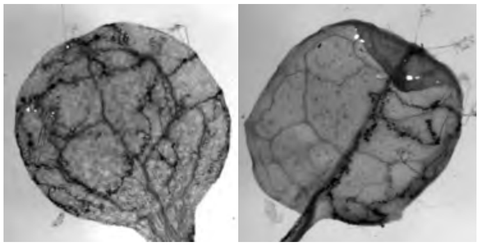

Br-0

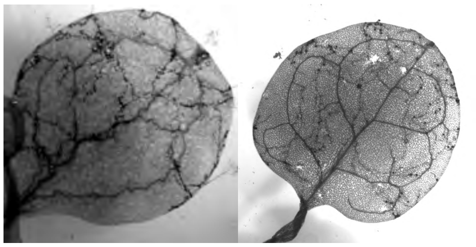

Bur-0

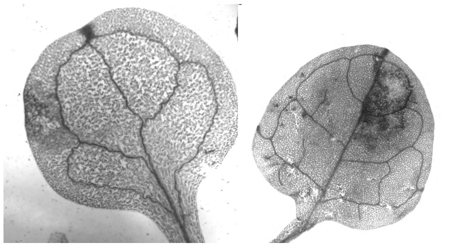

Cotyledon | True leaf

C24

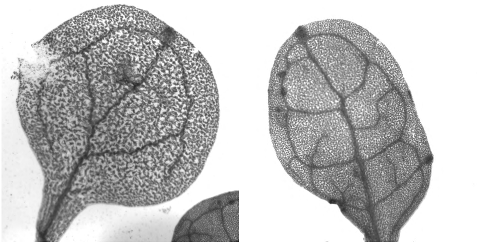

CIBC-5

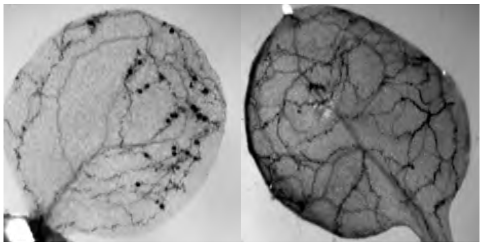

Col-0

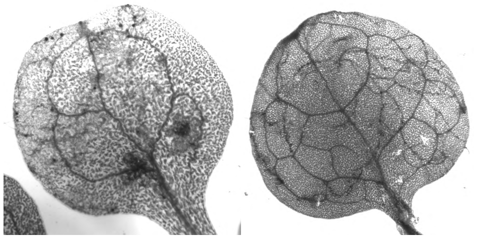

Ct-1

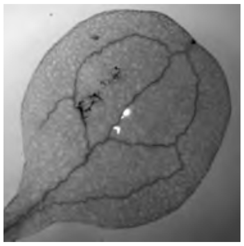

Cvi-0

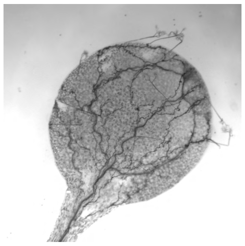

Eden-1

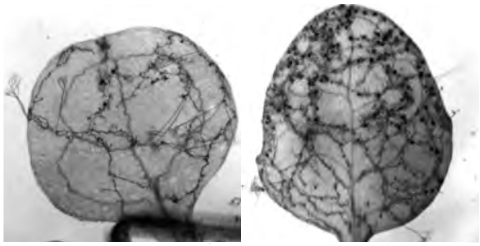

Edi-0

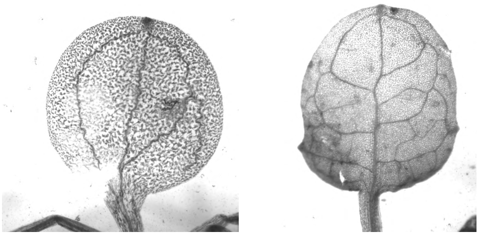

Supplemental Dataset 2. *Hpa* Maks9

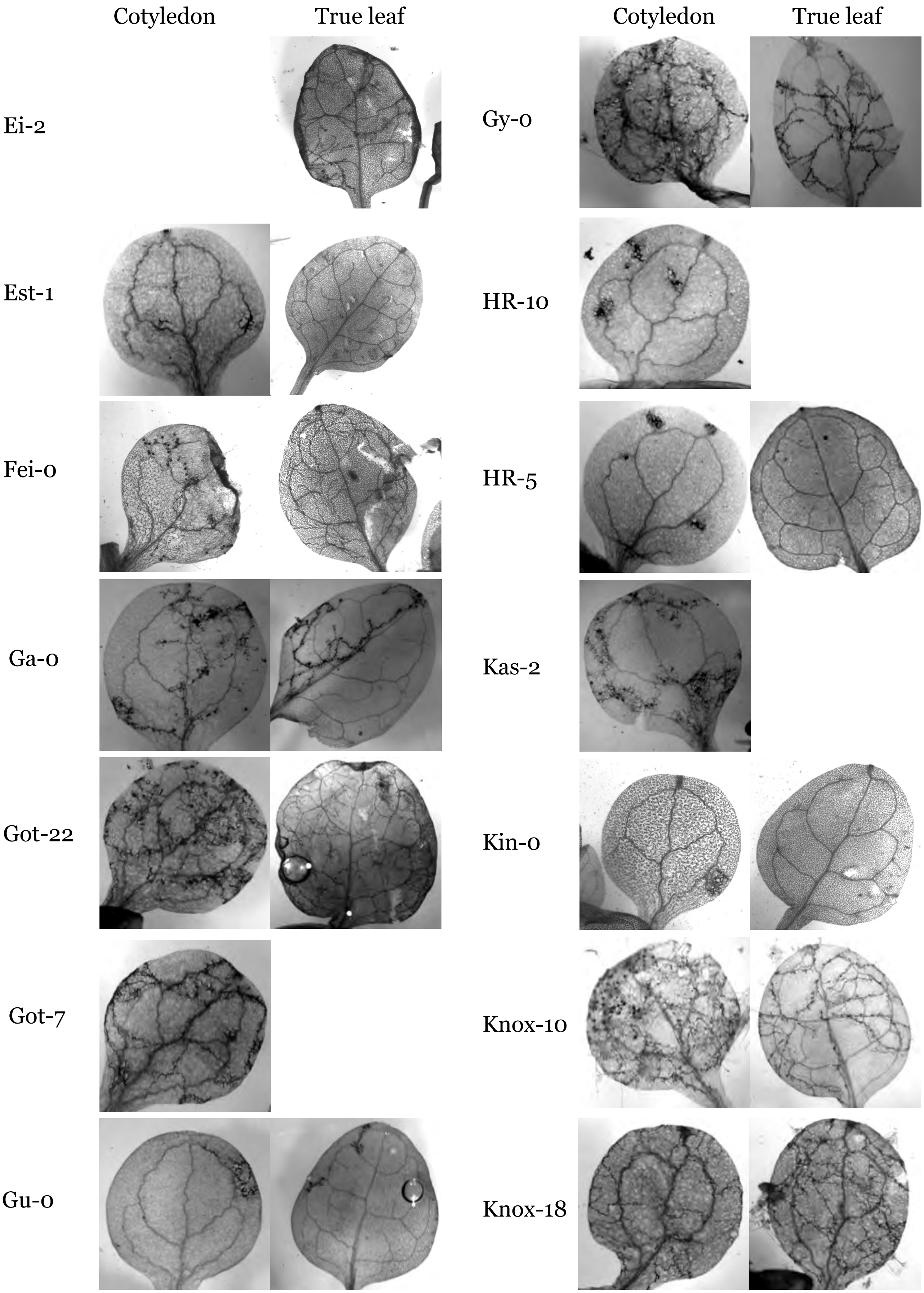

Supplemental Dataset 2. *Hpa* Maks9

Cotyledon | True leaf

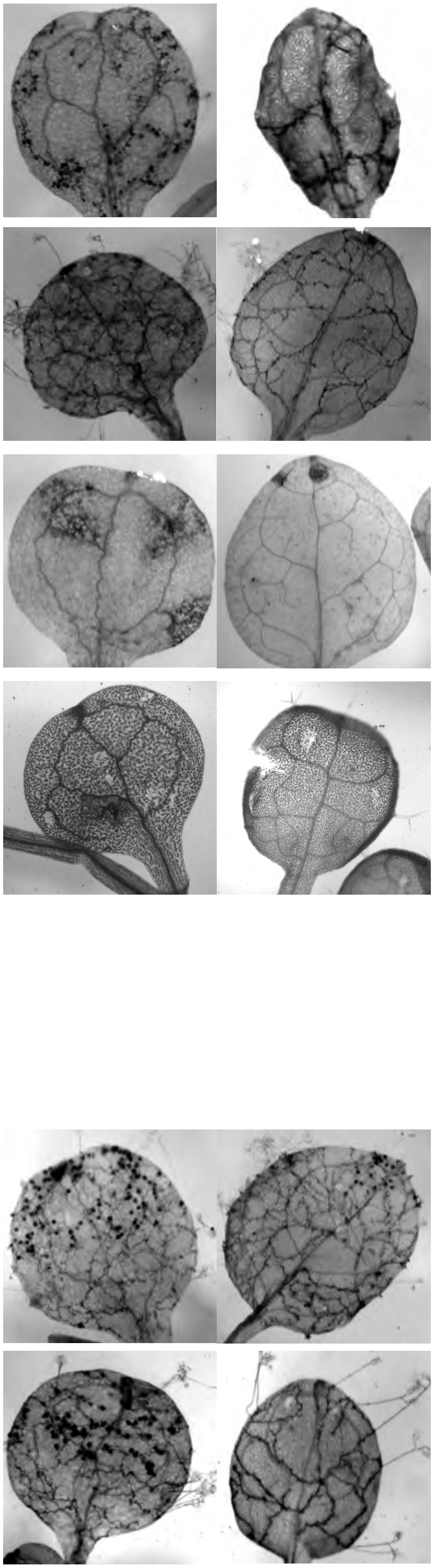

Kondara

Kz-1

Kz-9

Ler-1

LL-0

Lov-1

Lov-5

Cotyledon | True leaf

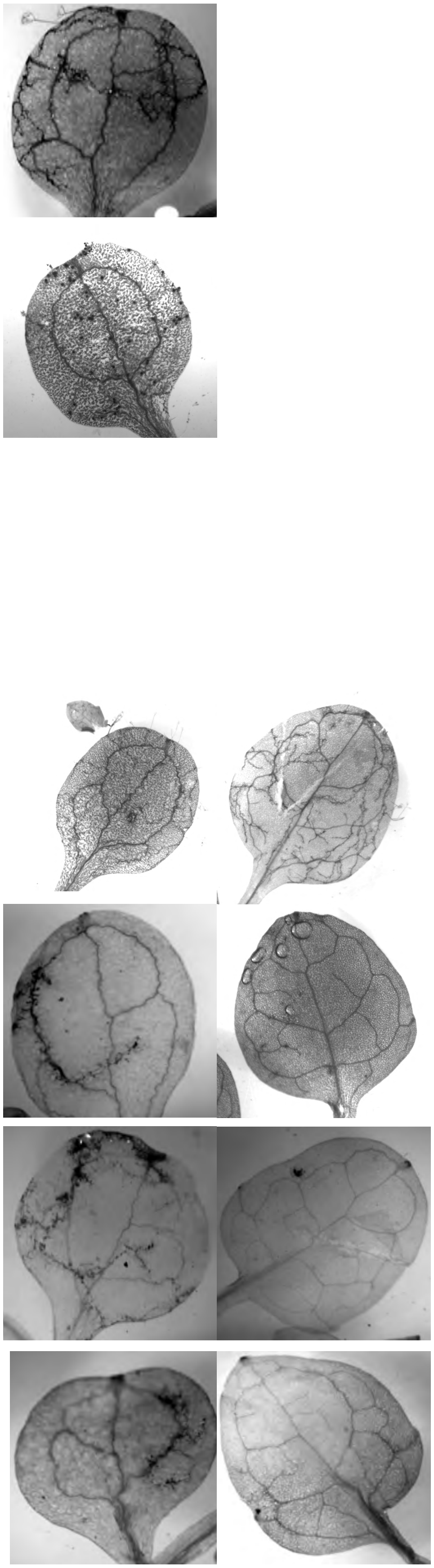

Lp2-2

Lz-0

Mr-0

Mrk-0

Ms-0

Mt-0

Mz-0

Supplemental Dataset 2. *Hpa* Maks9

Cotyledon | True leaf

N13

Nd-1

NFA-8

NFA-10

Nok-3

Omo2-3

Oy-0

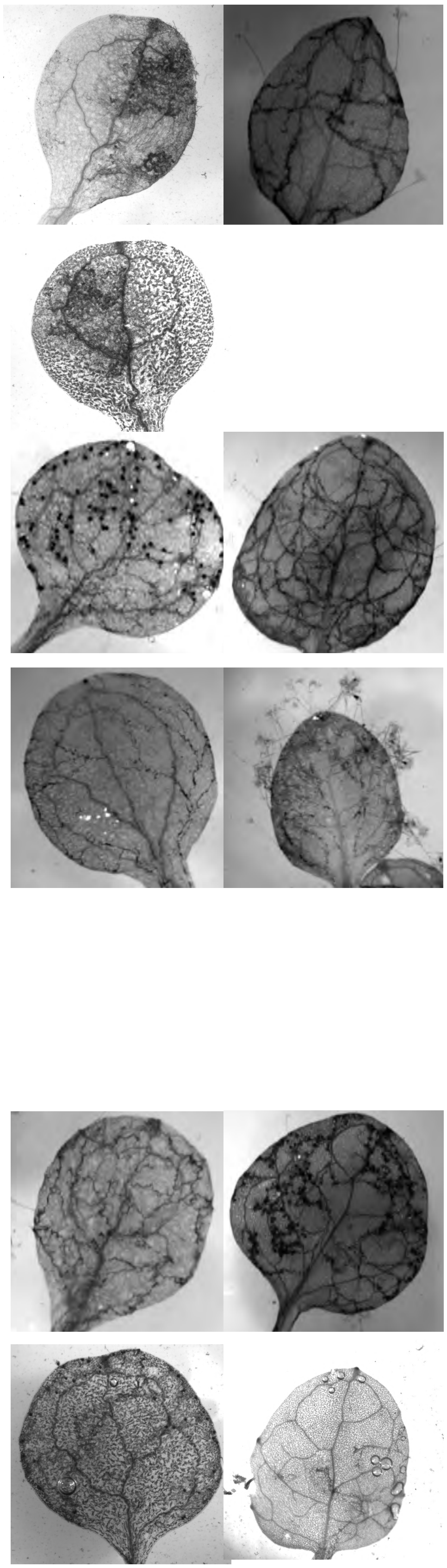

Cotyledon | True leaf

Pna-17

Pna-10

Pro-0

Pu2-23

Pu2-7

Ra-0

Ren-1

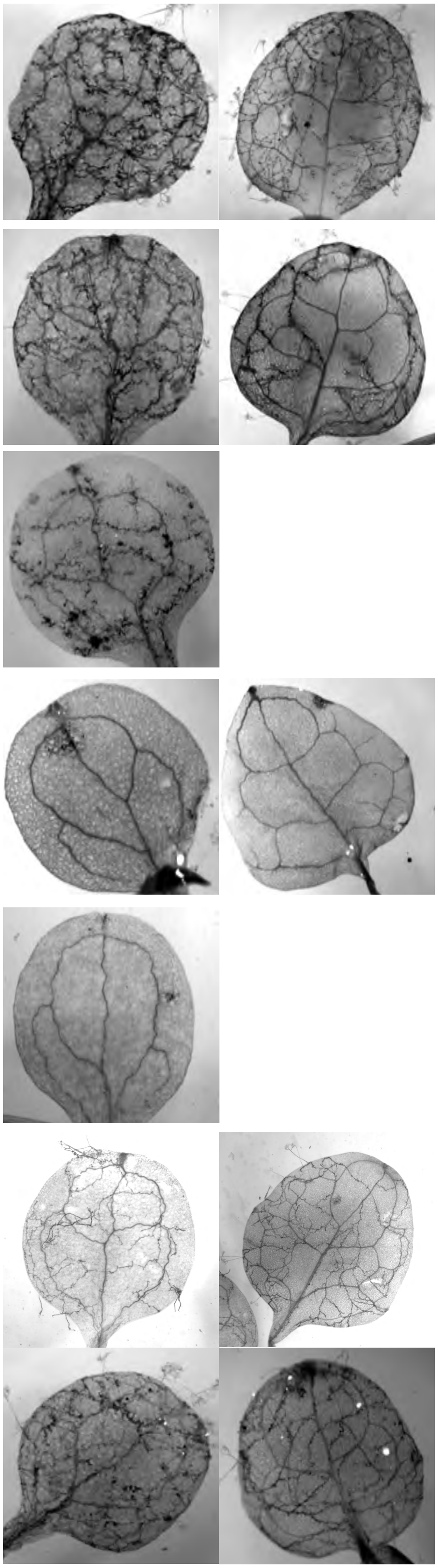

Supplemental Dataset 2. *Hpa* Maks9

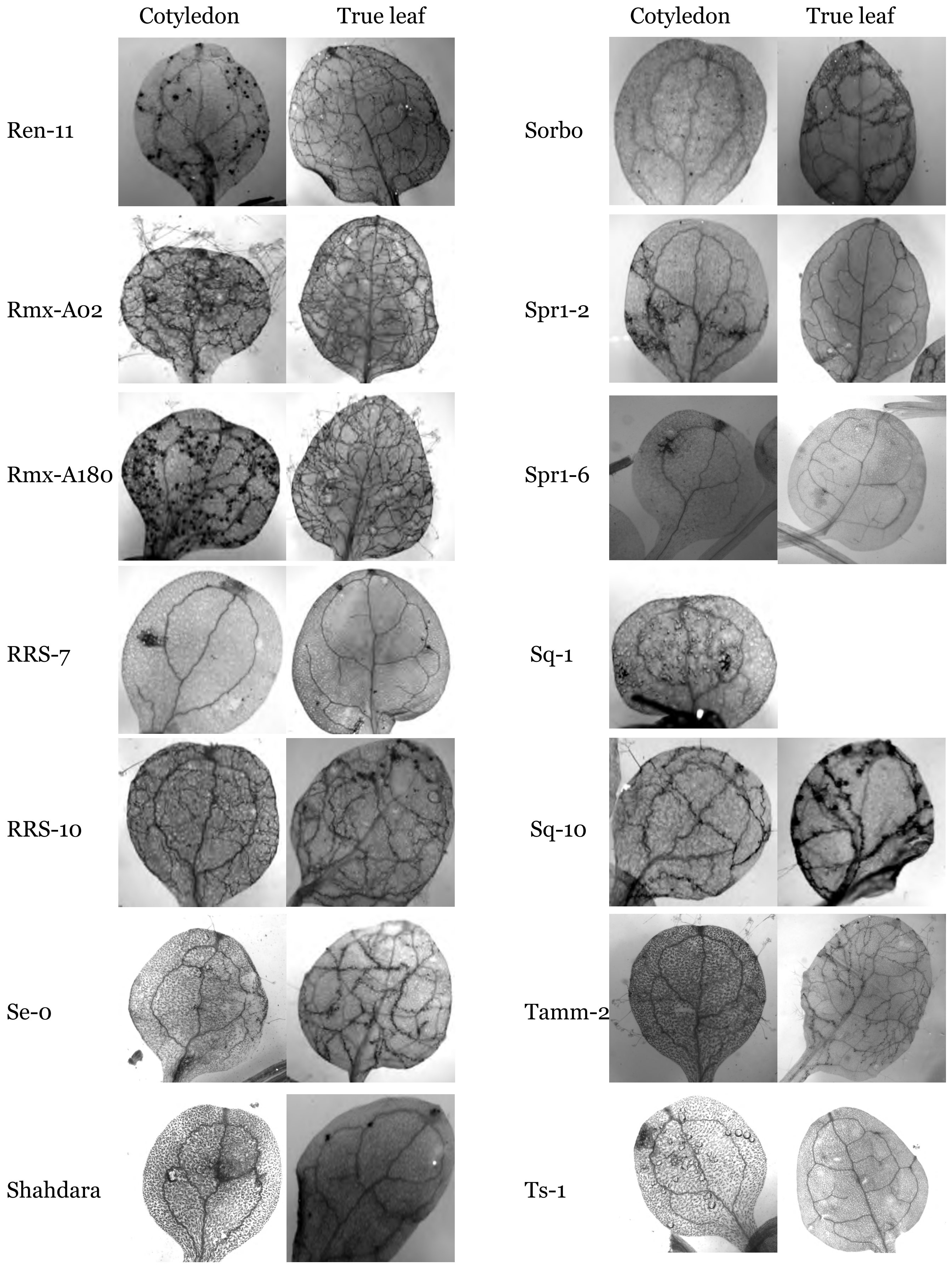

Supplemental Dataset 2. *Hpa* Maks9

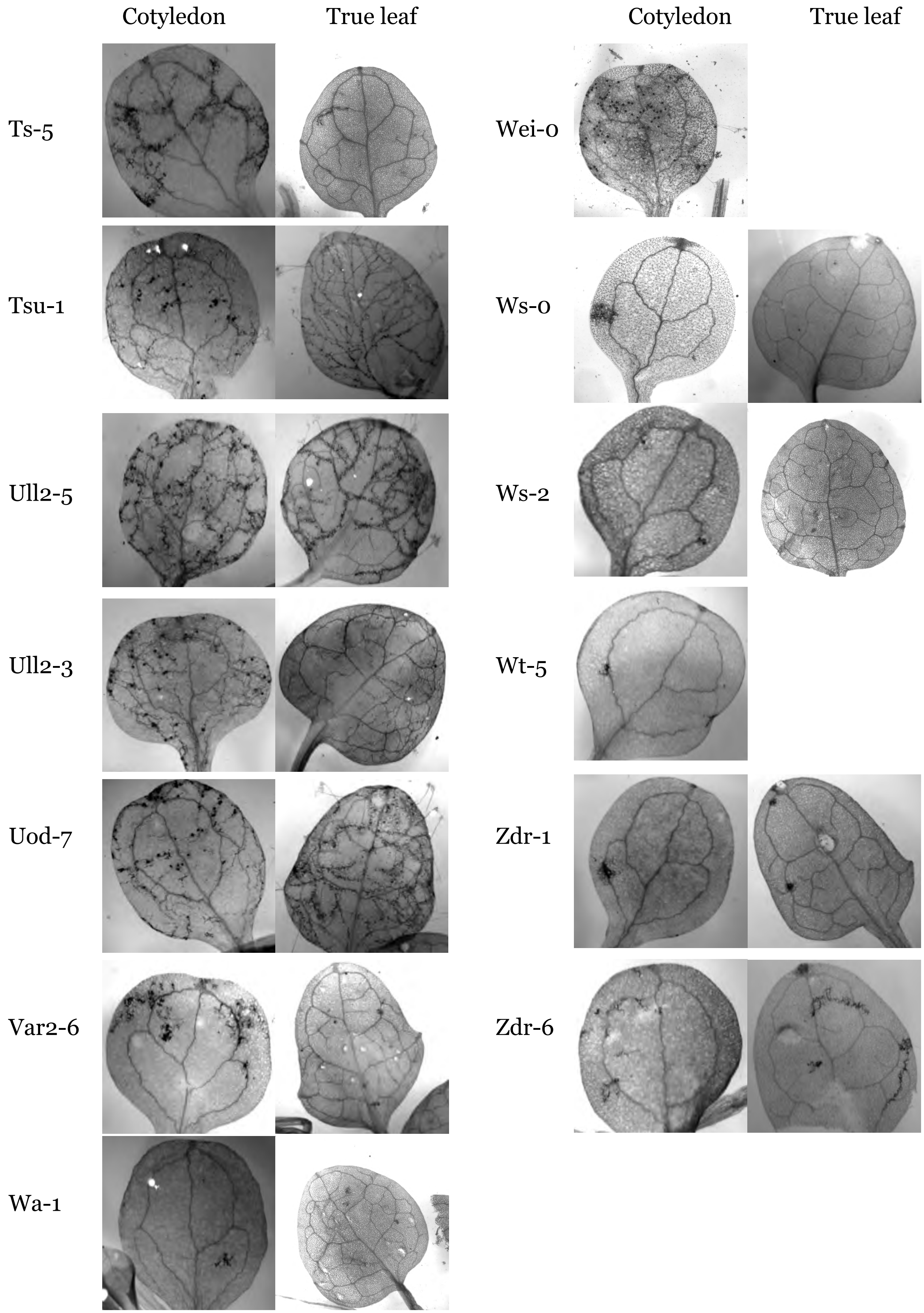

Supplement: Dataset S2 — Images of the trypan blue-stained Arabidopsis cotyledons and true leaves inoculated with Hpa Maks9. (PDF) [file pone.0028765.s005.pdf]
