# Supplementary material for: Global Analysis of Arabidopsis/Downy Mildew Interactions Reveals Prevalence of Incomplete Resistance and Rapid Evolution of Pathogen Recognition
Source: PLoS One. 2011 Dec 14;6(12):e28765. doi: 10.1371/journal.pone.0028765 (PMC3237489; doi:10.1371/journal.pone.0028765)

Supplemental Dataset 3. *Hpa* Emco5

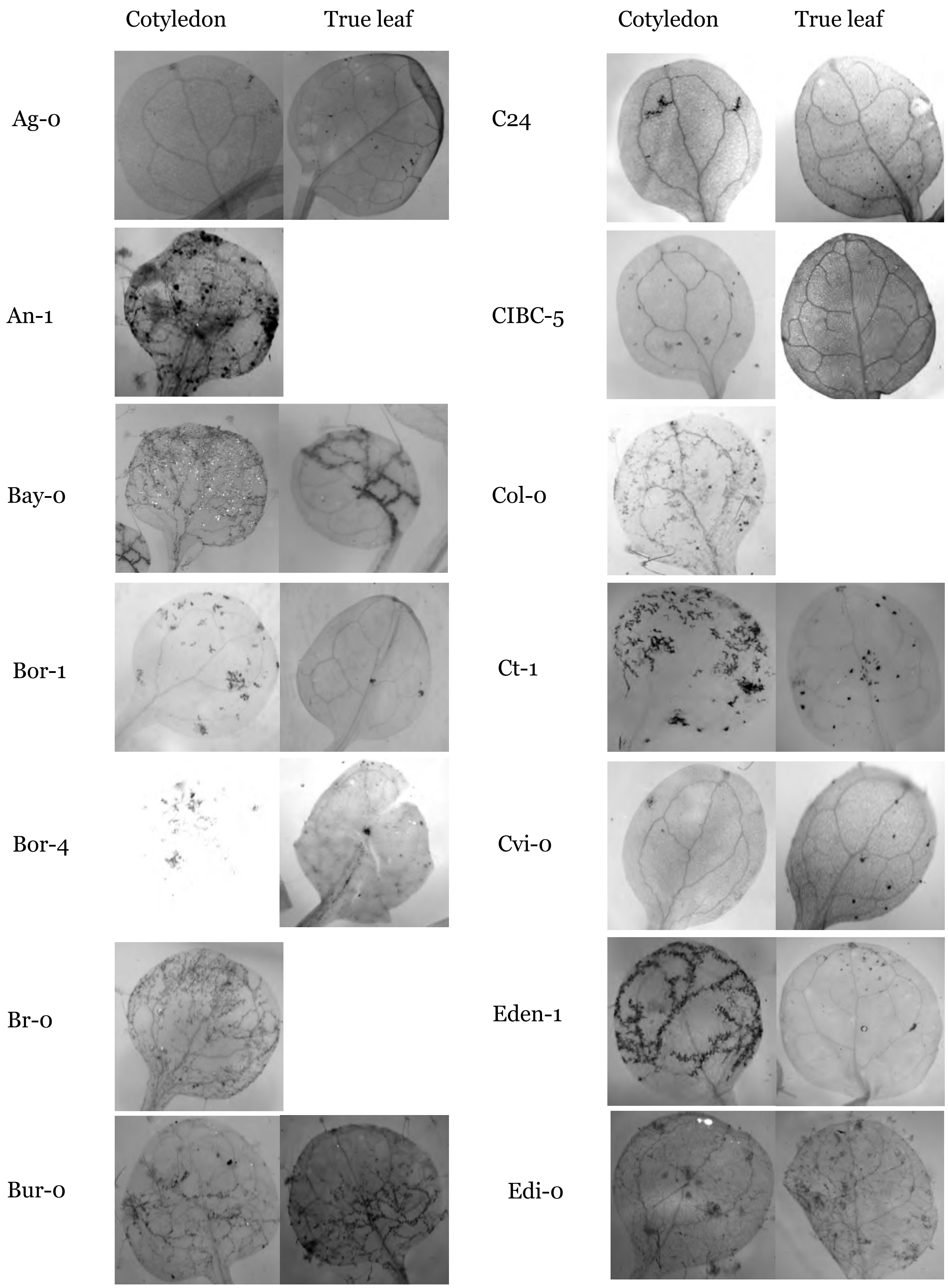

Supplemental Dataset 3. *Hpa* Emco5

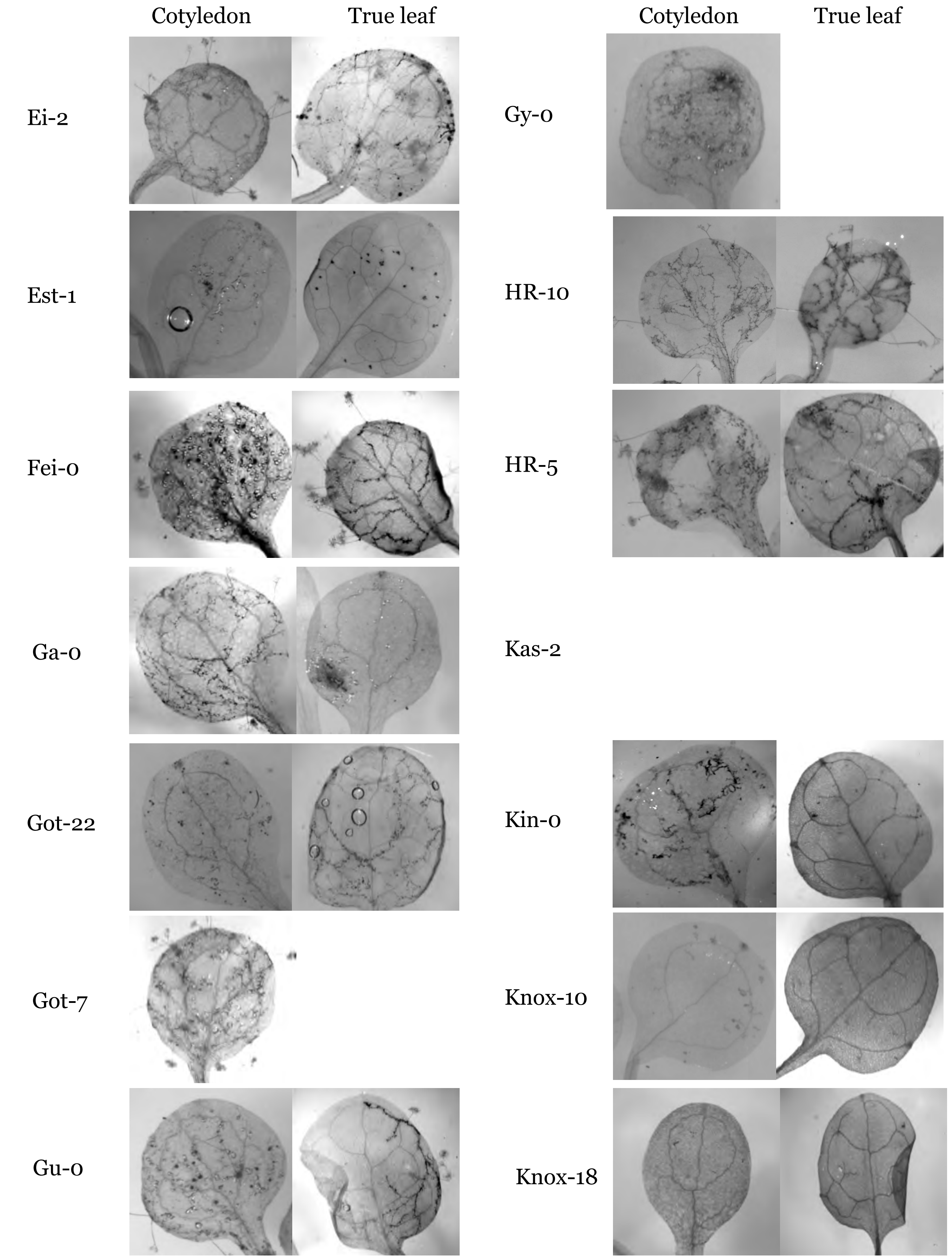

Supplemental Dataset 3. *Hpa* Emco5

Cotyledon | True leaf

Kondara

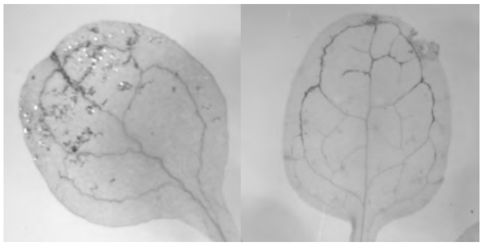

Kz-1

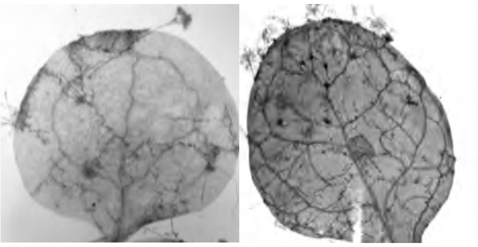

Kz-9

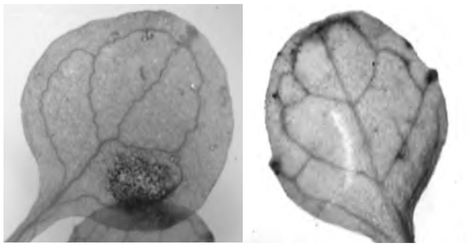

Ler-1

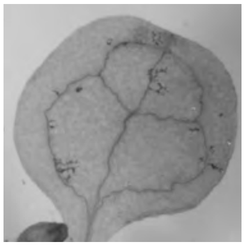

LL-0

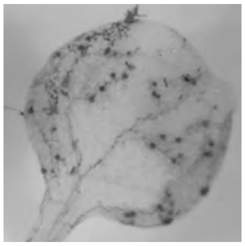

Lov-1

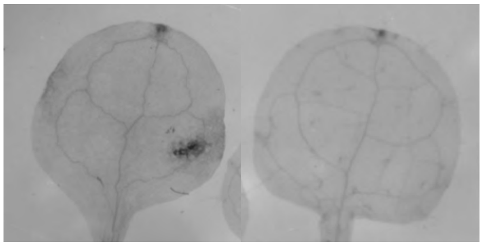

Lov-5

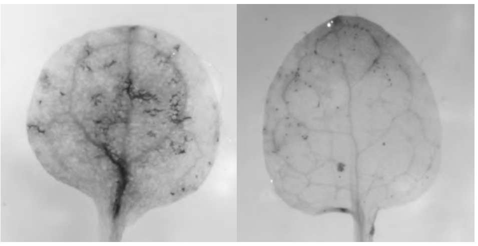

Cotyledon | True leaf

Lp2-2

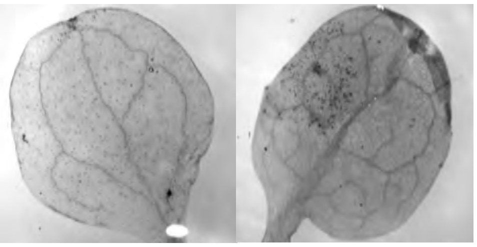

Lz-0

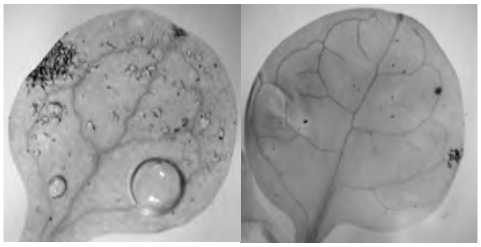

Mr-0

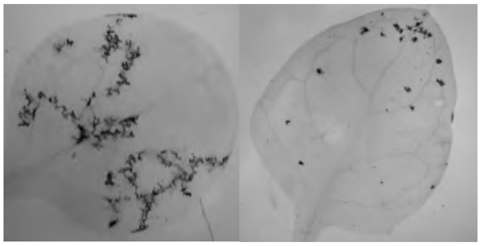

Mrk-0

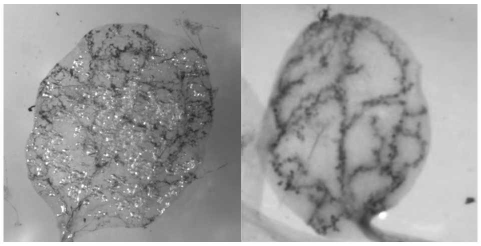

Ms-0

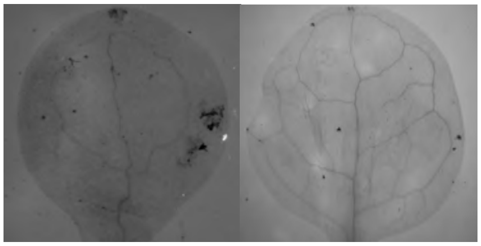

Mt-0

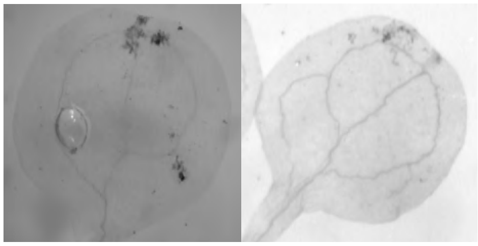

Mz-0

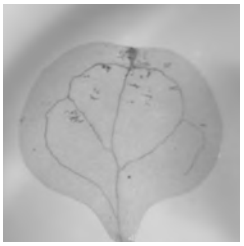

Supplemental Dataset 3. *Hpa* Emco5

Cotyledon | True leaf

N13

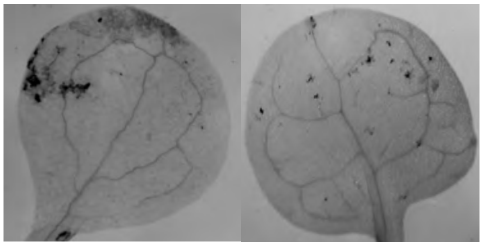

Nd-1

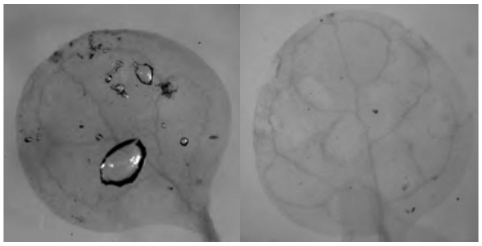

NFA-8

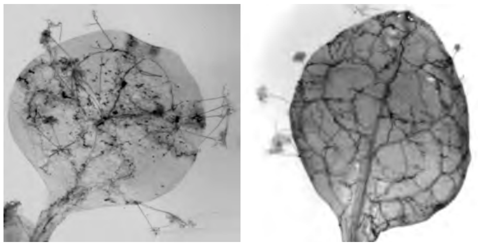

NFA-10

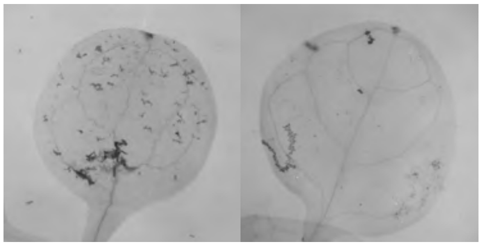

Nok-3

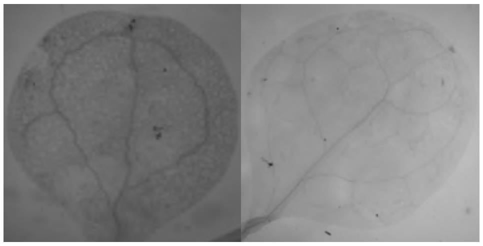

Omo2-3

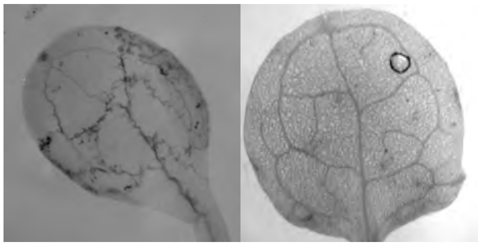

Oy-0

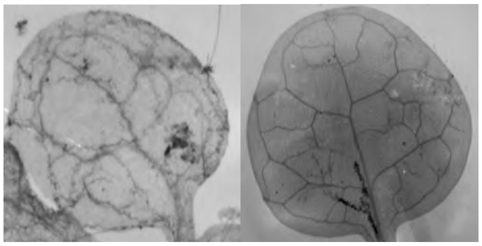

Cotyledon | True leaf

Pna-17

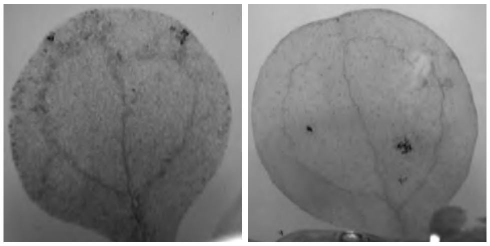

Pna-10

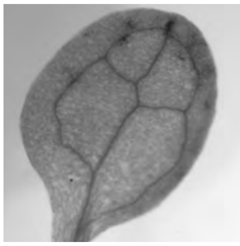

Pro-0

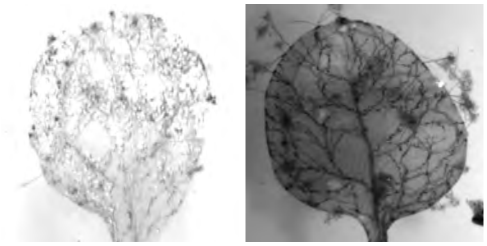

Pu2-23

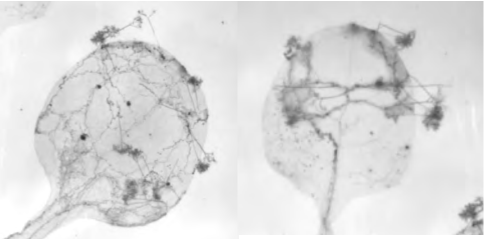

Pu2-7

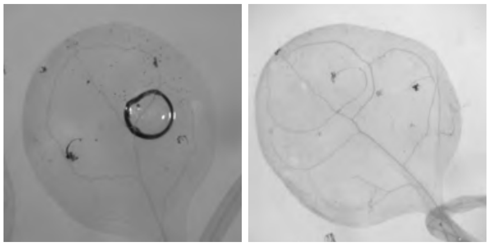

Ra-0

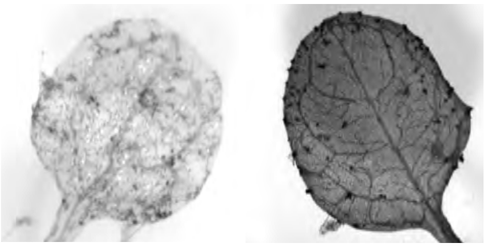

Ren-1

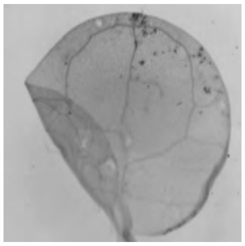

Supplemental Dataset 3. *Hpa* Emco5

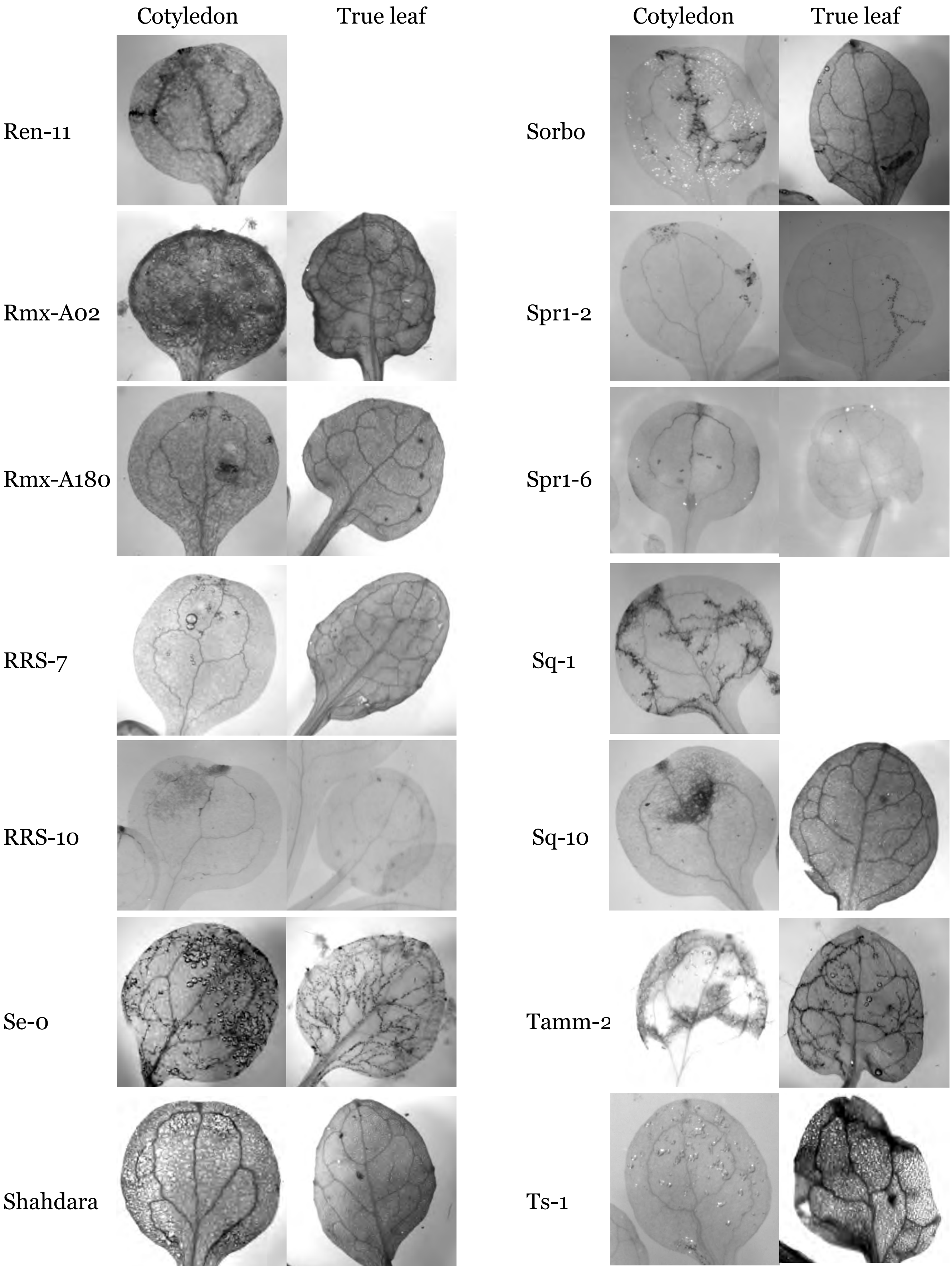

Supplemental Dataset 3. *Hpa* Emco5

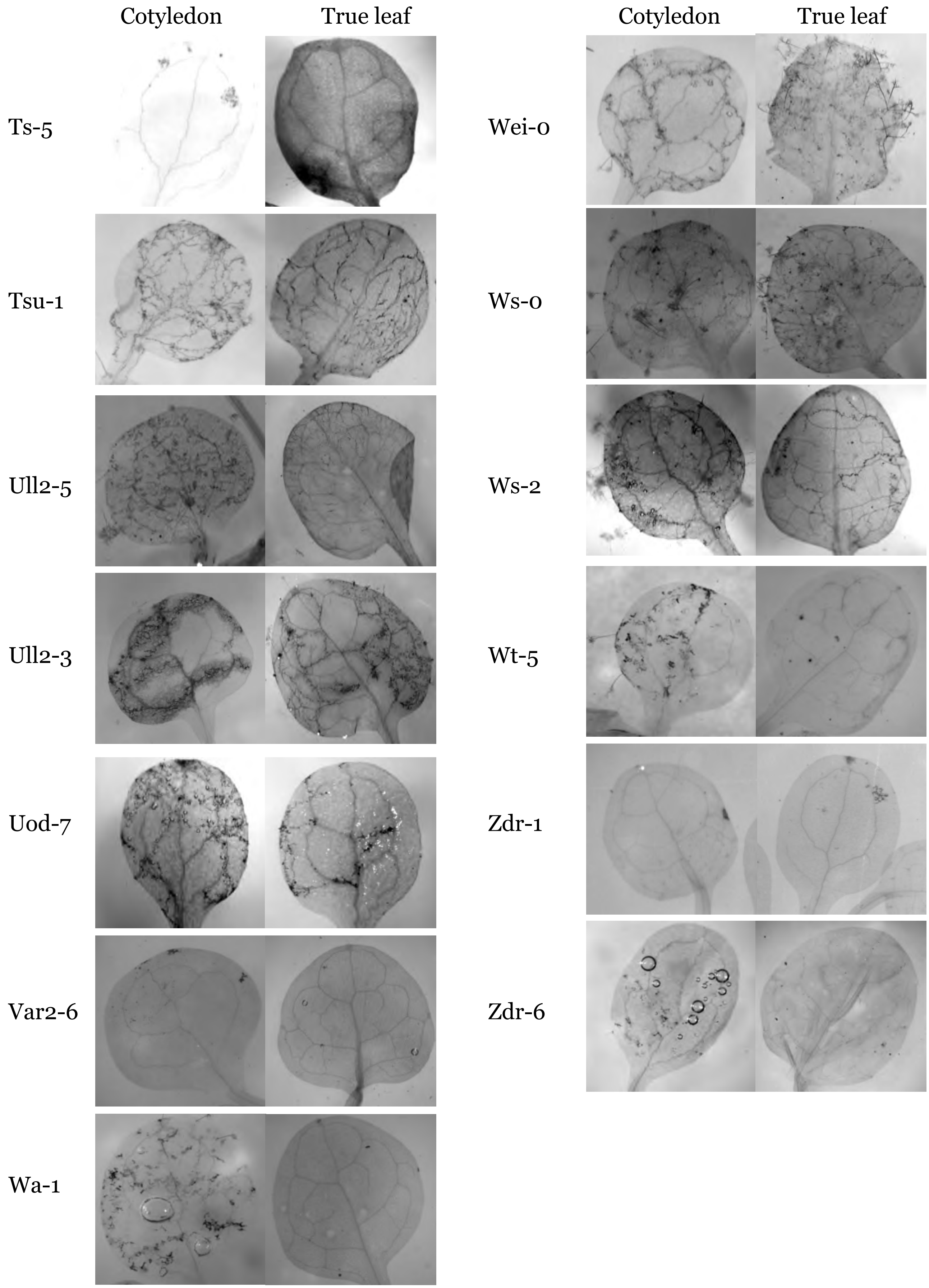

Supplement: Dataset S3 — Images of the trypan blue-stained Arabidopsis cotyledons and true leaves inoculated with Hpa Emco5. (PDF) [file pone.0028765.s006.pdf]
