# Supplementary material for: Global Analysis of Arabidopsis/Downy Mildew Interactions Reveals Prevalence of Incomplete Resistance and Rapid Evolution of Pathogen Recognition
Source: PLoS One. 2011 Dec 14;6(12):e28765. doi: 10.1371/journal.pone.0028765 (PMC3237489; doi:10.1371/journal.pone.0028765)

Supplemental Dataset 4. *Hpa* Cala2

	Cotyledon	True leaf		Cotyledon	True leaf
Ag-0	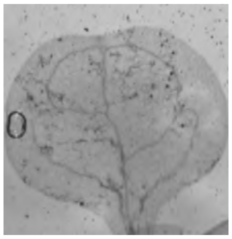	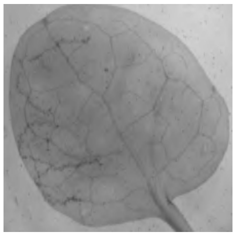	C24	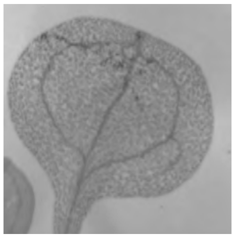	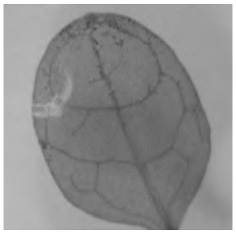
An-1	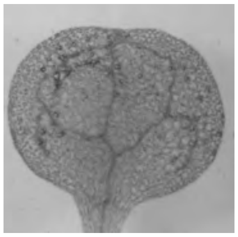	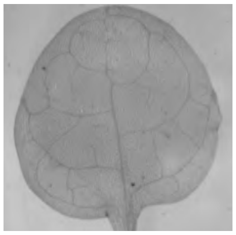	CIBC-5	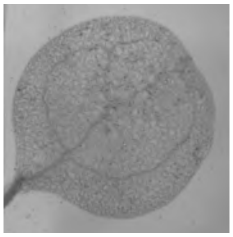	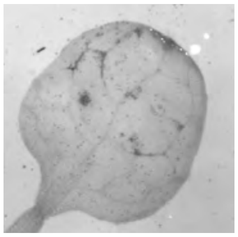
Bay-0	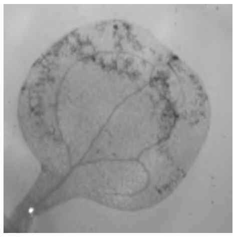	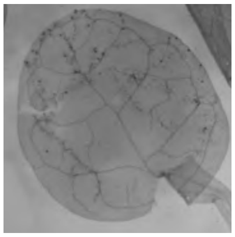	Col-0	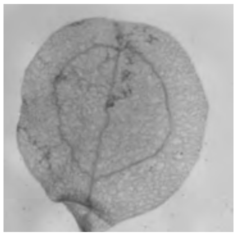	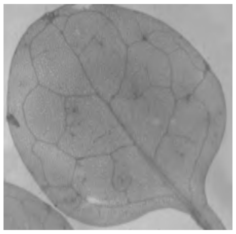
Bor-1	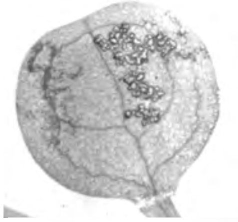	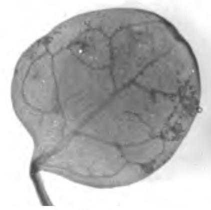	Ct-1	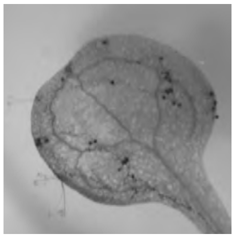	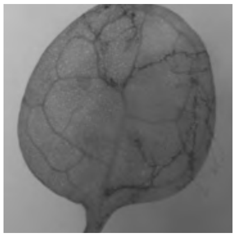
Bor-4	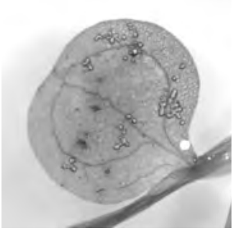	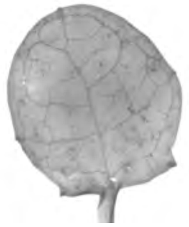	Cvi-0	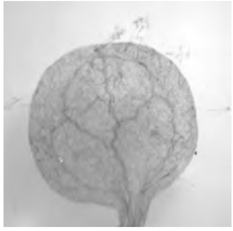	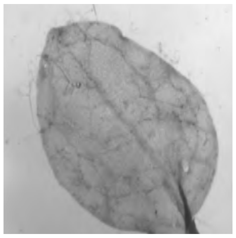
Br-0	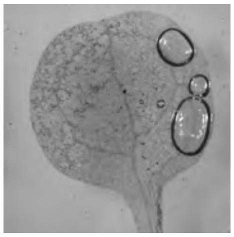	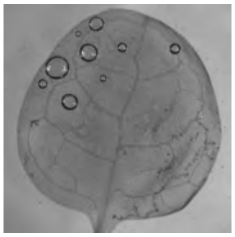	Eden-1	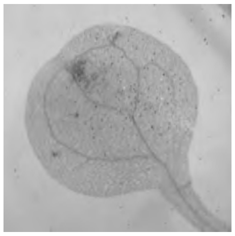	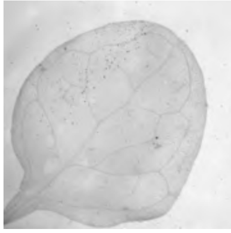
Bur-0	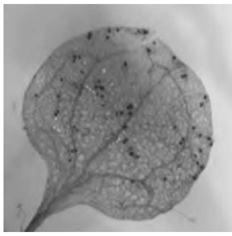	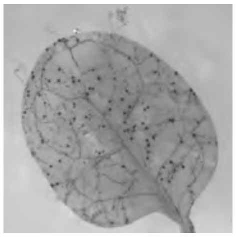	Edi-0	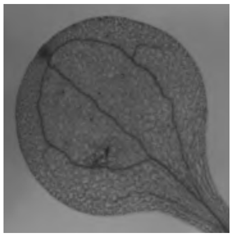	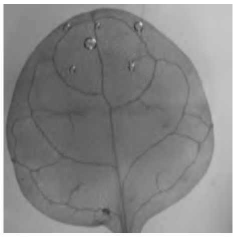

Supplemental Dataset 4. *Hpa* Cala2

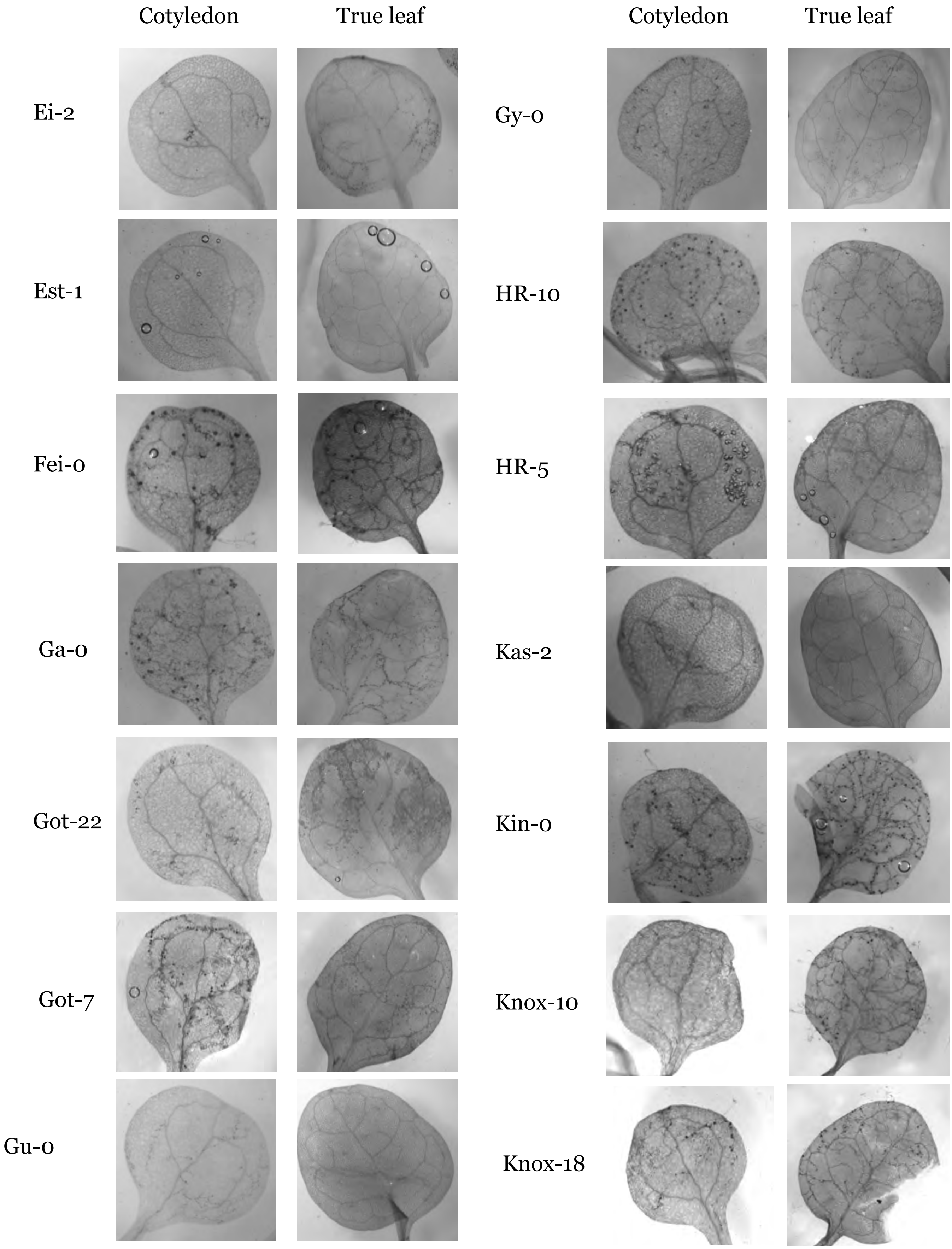

Supplemental Dataset 4. *Hpa* Cala2

Cotyledon | True leaf

Kondara
Kz-1
Kz-9
Ler-1
LL-0
Lov-1
Lov-5

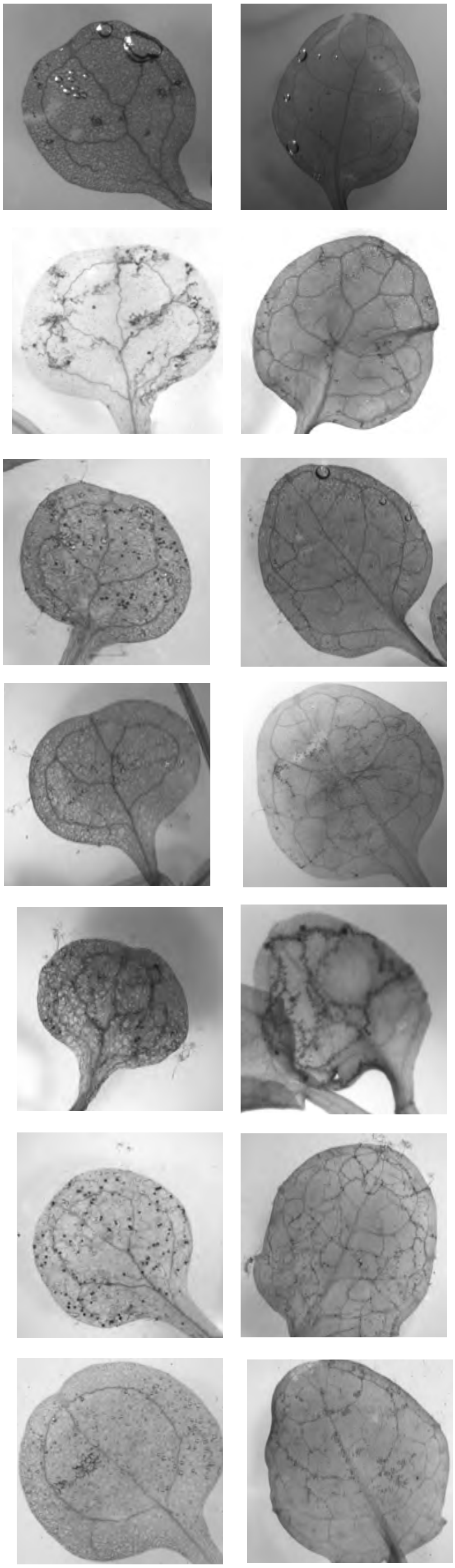

Cotyledon | True leaf

Lp2-2
Lz-0
Mr-0
Mrk-0
Ms-0
Mt-0
Mz-0

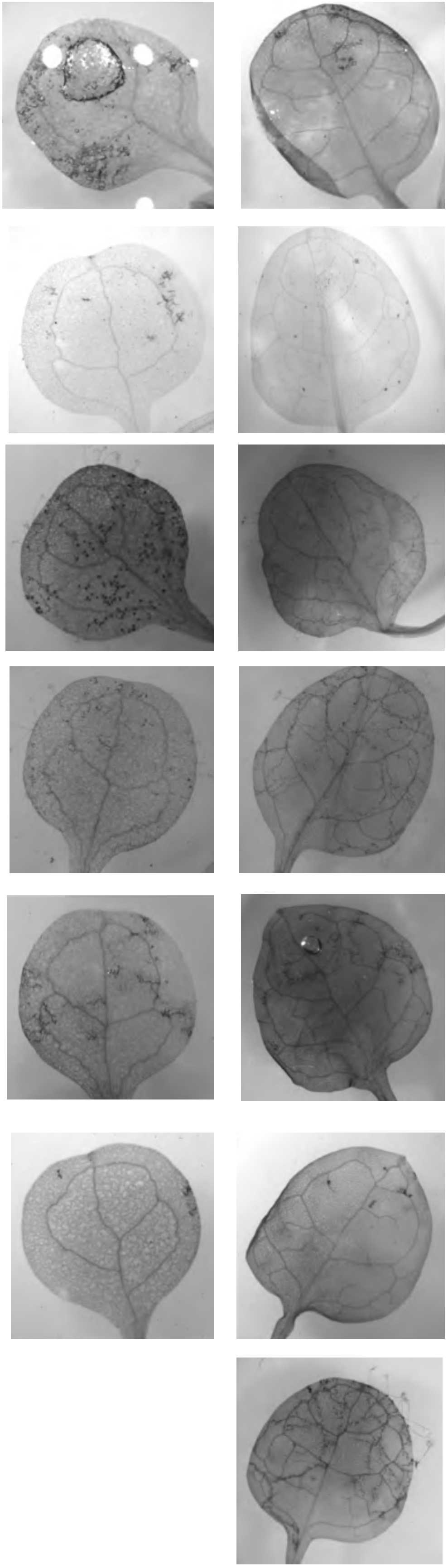

Supplemental Dataset 4. *Hpa* Cala2

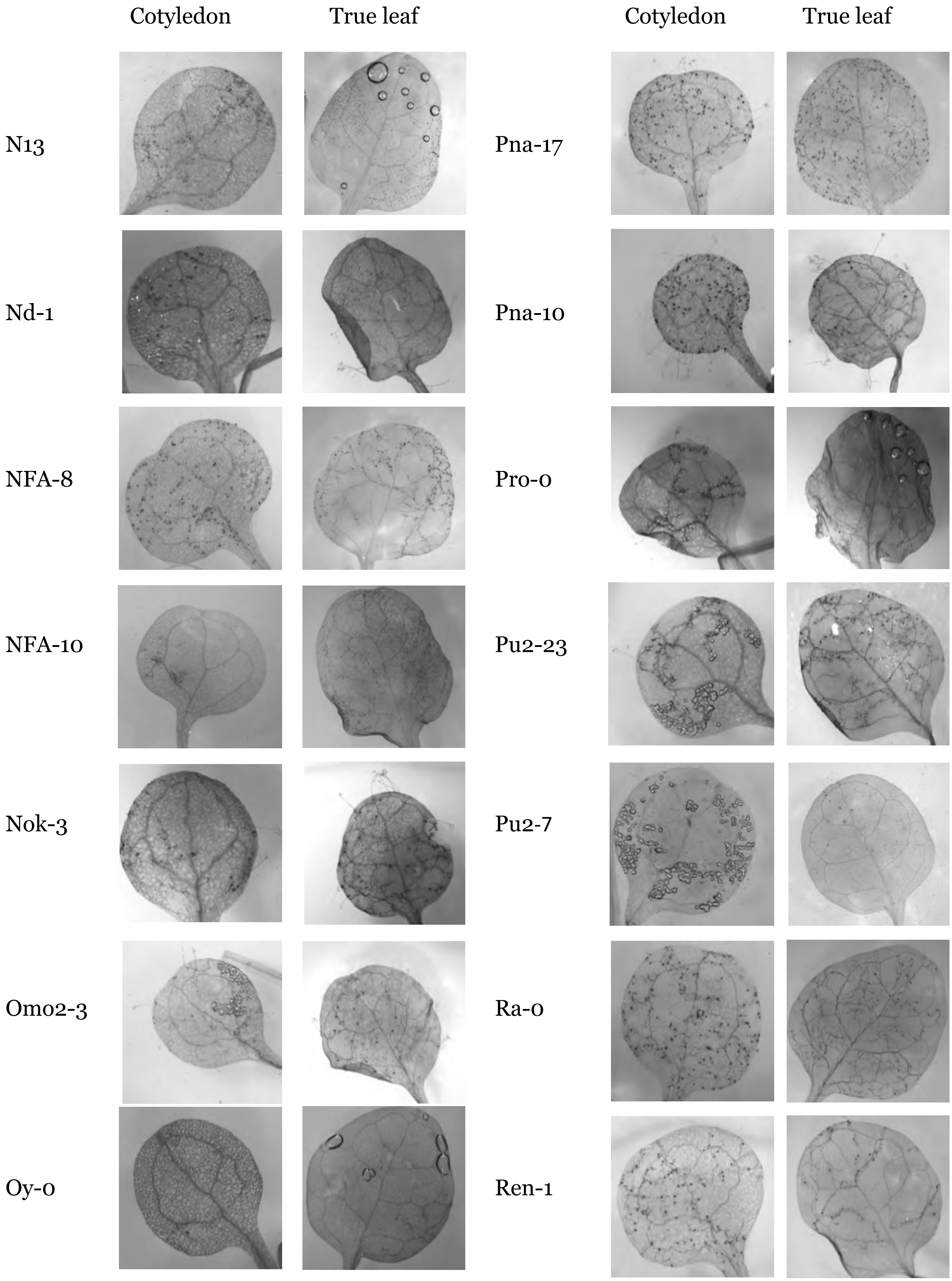

Supplemental Dataset 4. *Hpa* Cala2

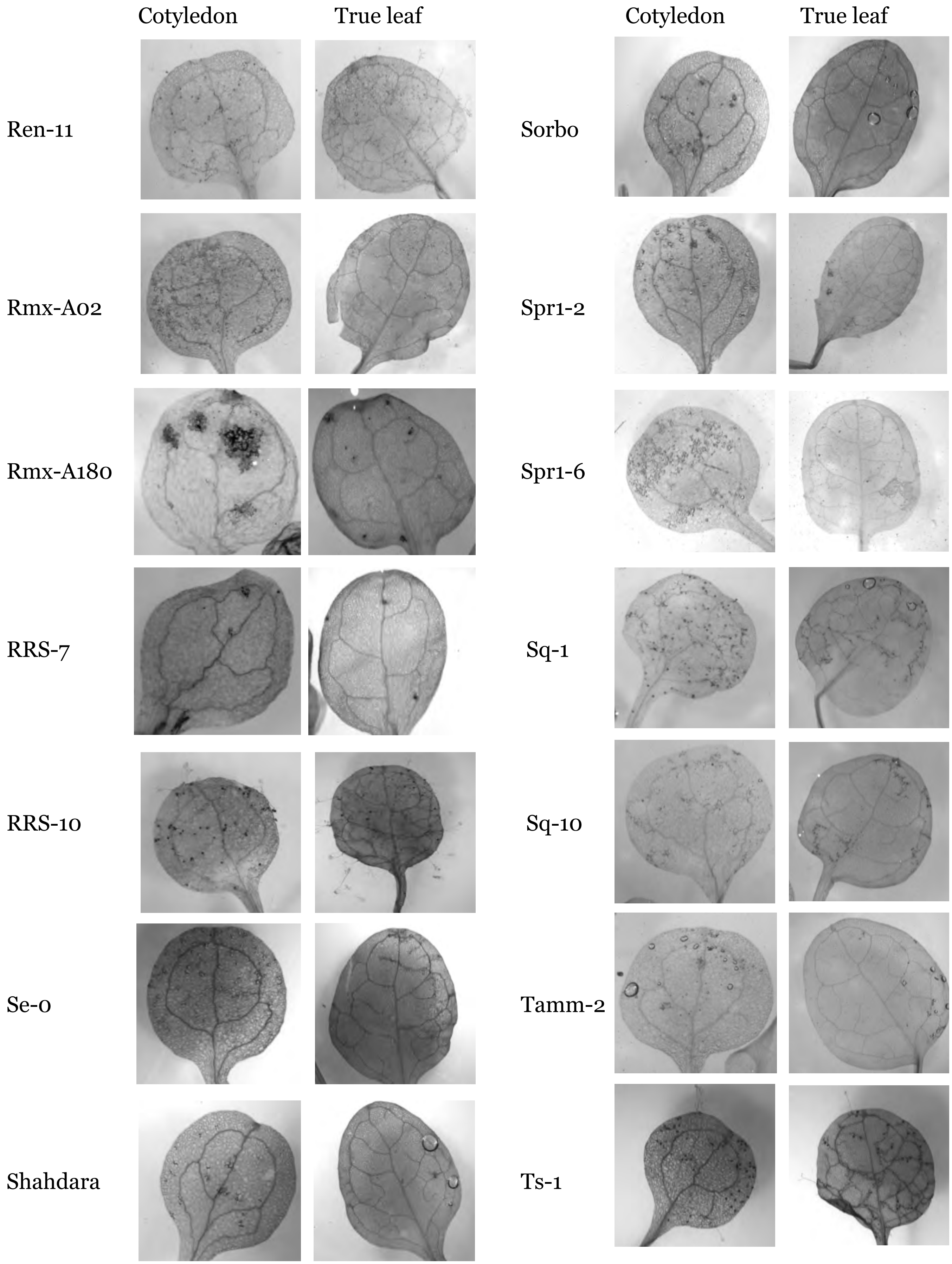

Supplemental Dataset 4. *Hpa* Cala2

	Cotyledon	True leaf

Ts-5

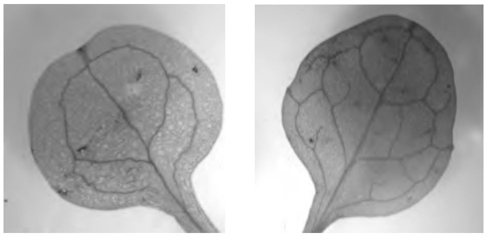

Tsu-1

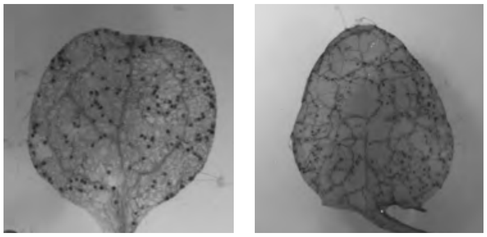

Ull2-5

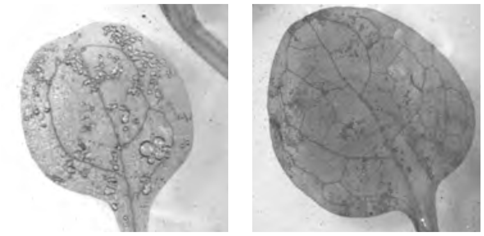

Ull2-3

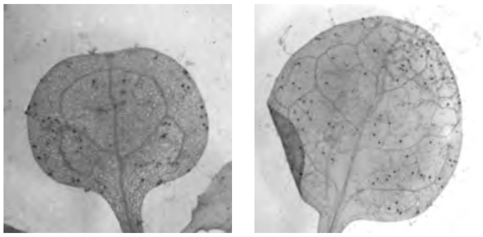

Uod-7

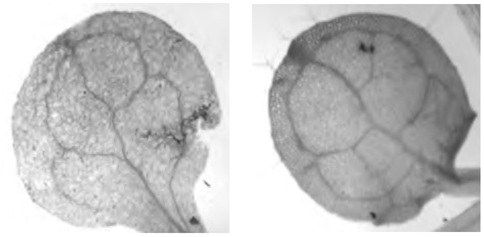

Var2-6

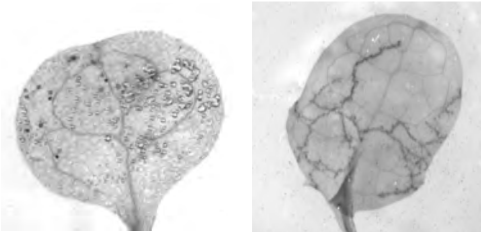

Wa-1

	Cotyledon	True leaf

Wei-0

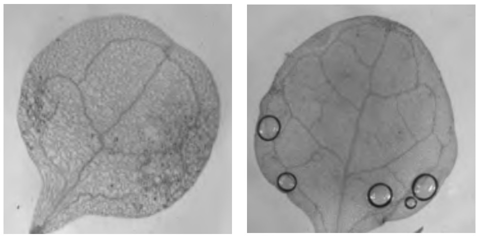

Ws-0

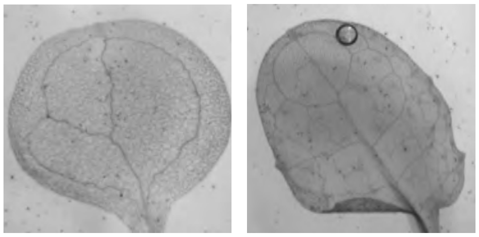

Ws-2

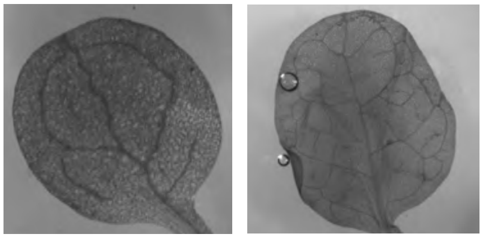

Wt-5

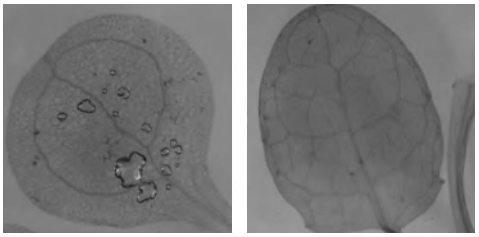

Zdr-1

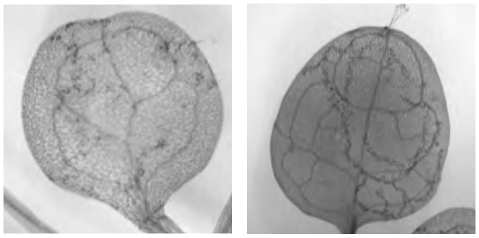

Zdr-6

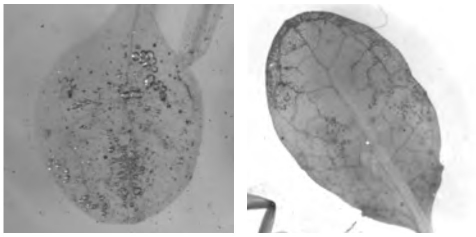

Supplement: Dataset S4 — Images of the trypan blue-stained Arabidopsis cotyledons and true leaves inoculated with Hpa Cala2. (PDF) [file pone.0028765.s007.pdf]
