# Supplementary material for: Global Analysis of Arabidopsis/Downy Mildew Interactions Reveals Prevalence of Incomplete Resistance and Rapid Evolution of Pathogen Recognition
Source: PLoS One. 2011 Dec 14;6(12):e28765. doi: 10.1371/journal.pone.0028765 (PMC3237489; doi:10.1371/journal.pone.0028765)

Supplemental Dataset 5. ***Hpa*** **Emwa1**

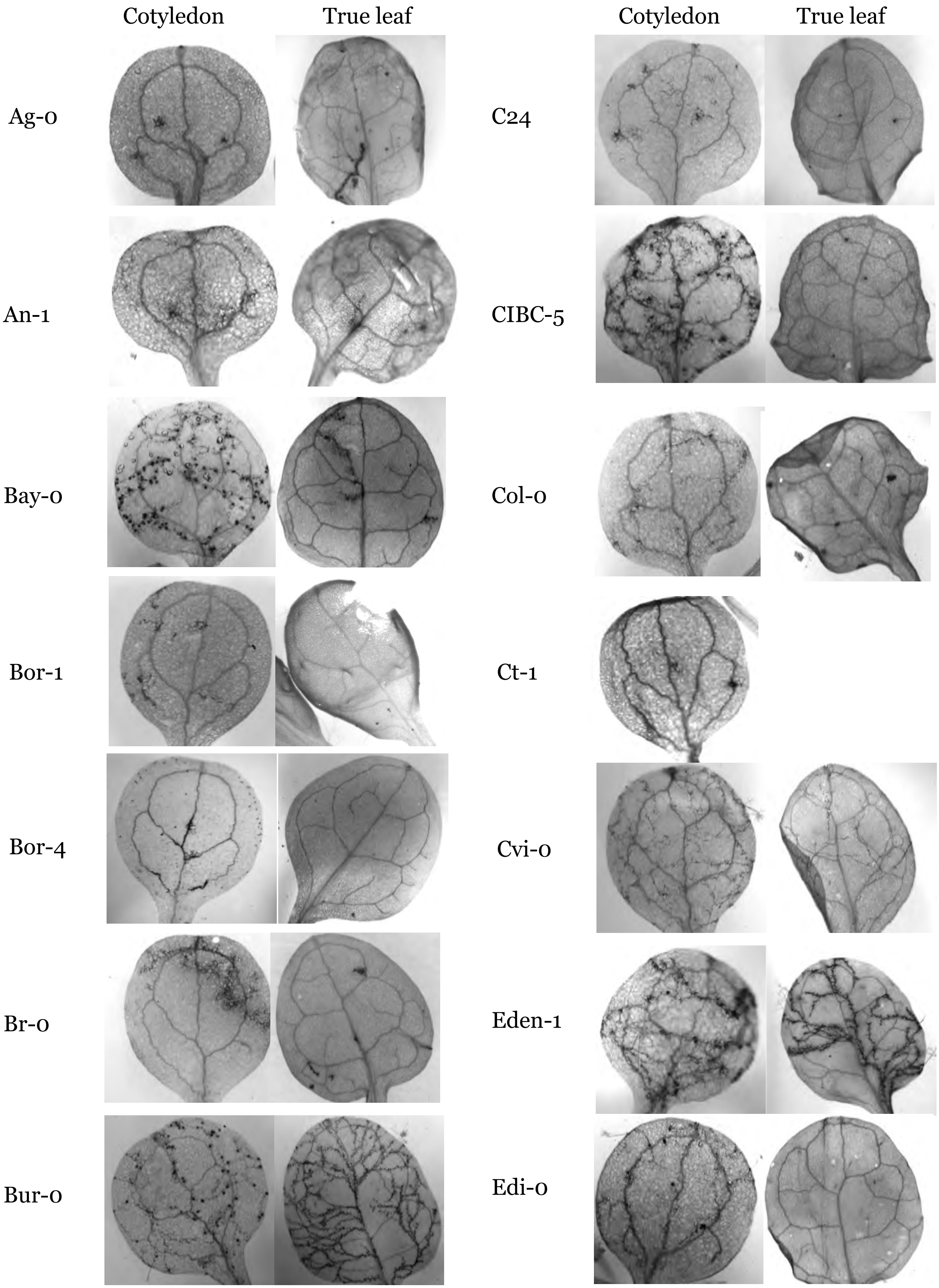

Supplemental Dataset 5. *Hpa* Emwa1

Cotyledon True leaf

Ei-2

Est-1

Fei-0

Ga-0

Got-22

Got-7

Gu-0

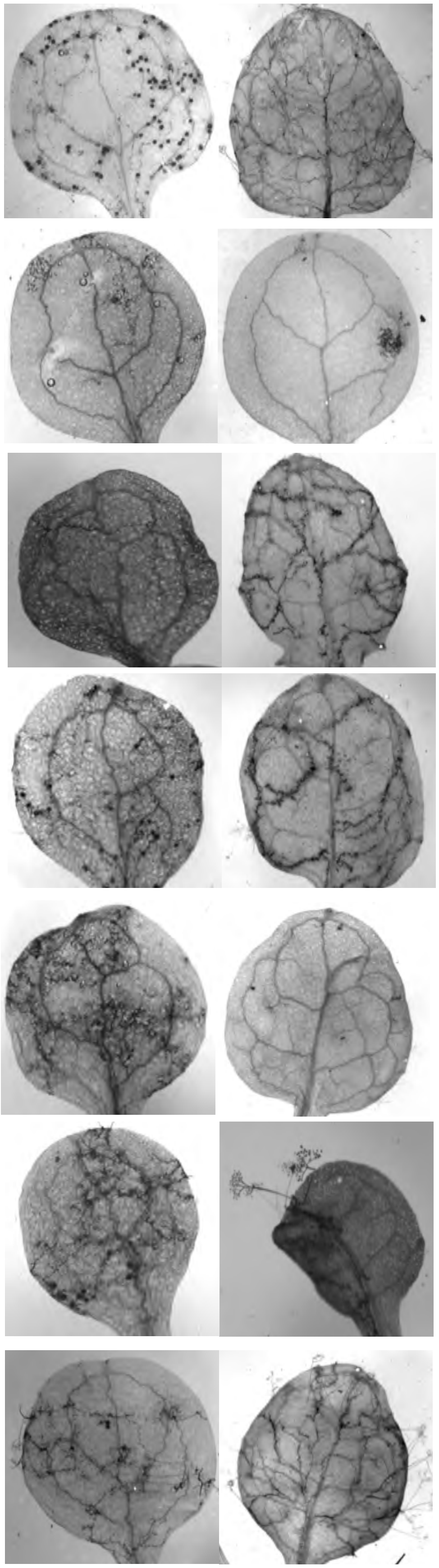

Cotyledon True leaf

Gy-0

HR-10

HR-5

Kas-2

Kin-0

Knox-10

Knox-18

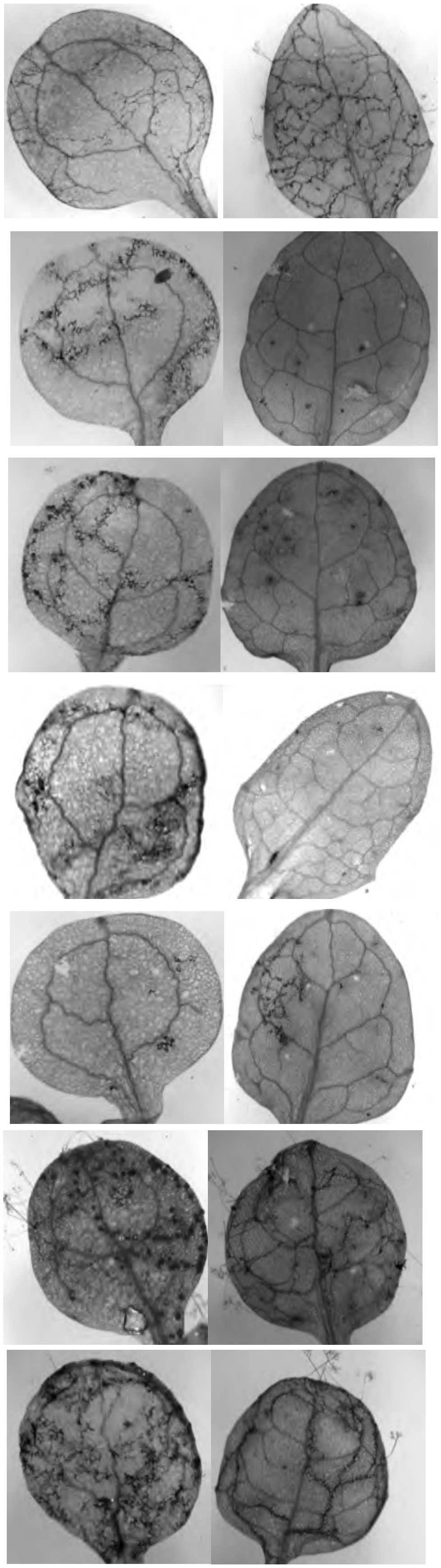

Supplemental Dataset 5. ***Hpa*** **Emwa1**

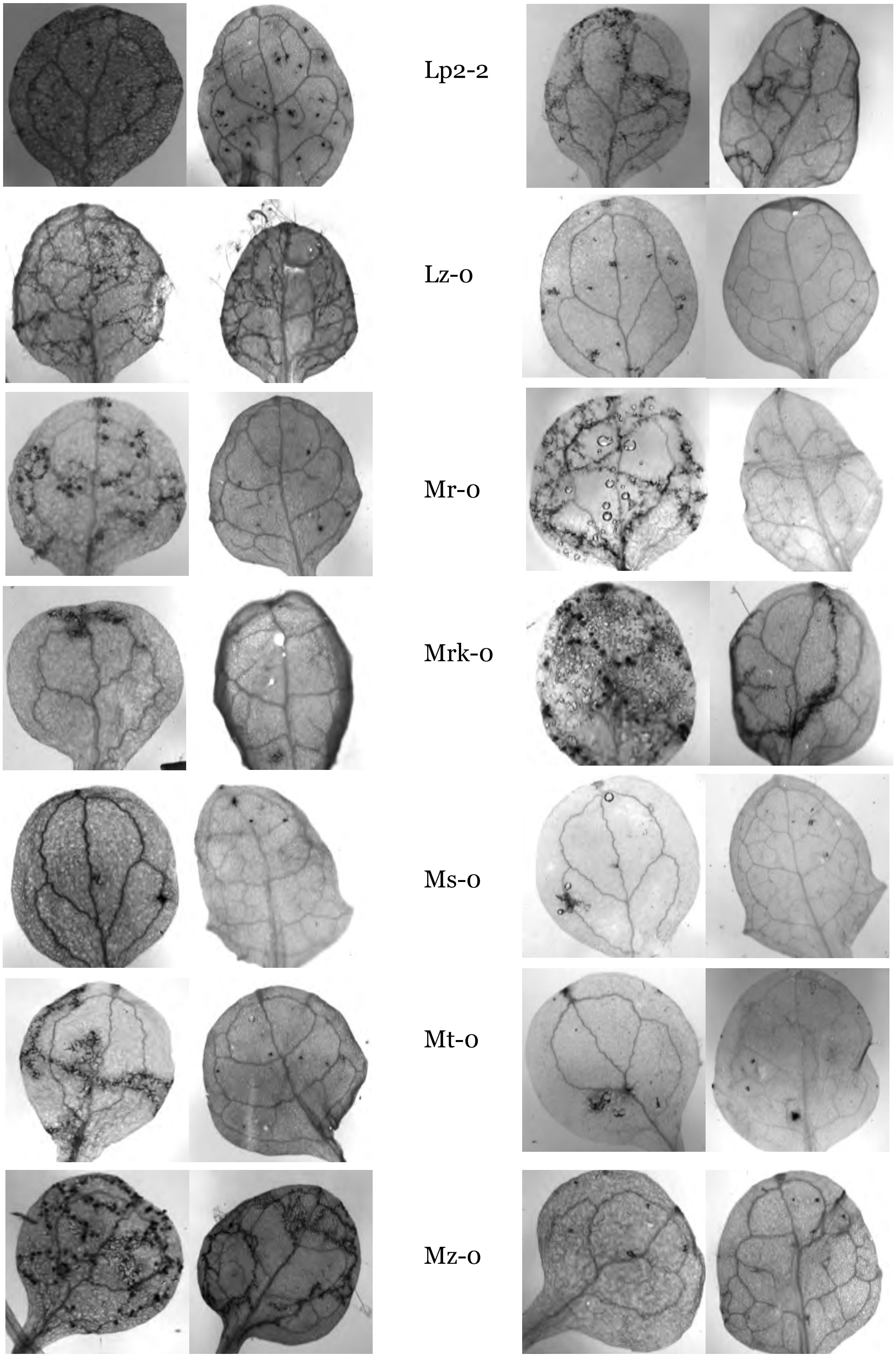

Cotyledon | True leaf | Cotyledon | True leaf

Kondara
Kz-1
Kz-9
Ler-1
LL-0
Lov-1
Lov-5
Lp2-2
Lz-0
Mr-0
Mrk-0
Ms-0
Mt-0
Mz-0

Supplemental Dataset 5. *Hpa* Emwa1

Cotyledon True leaf

N13

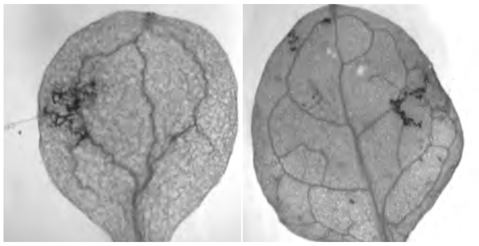

Nd-1

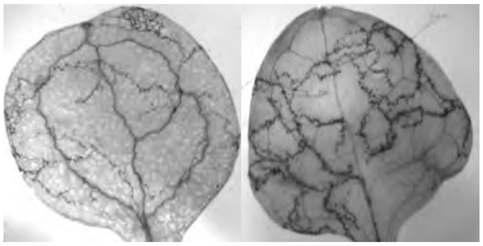

NFA-8

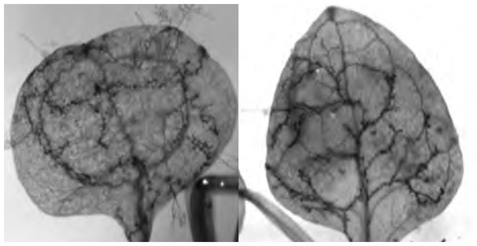

NFA-10

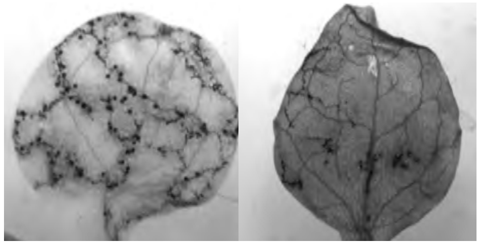

Nok-3

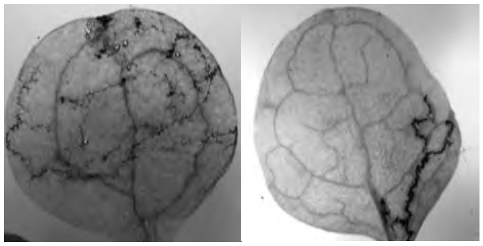

Omo2-3

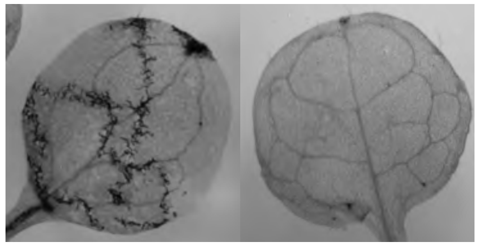

Oy-0

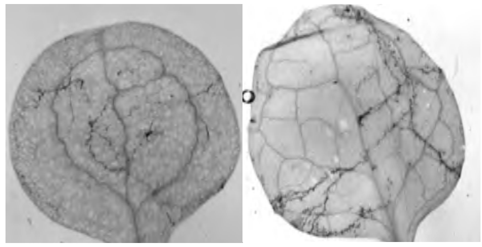

Cotyledon True leaf

Pna-17

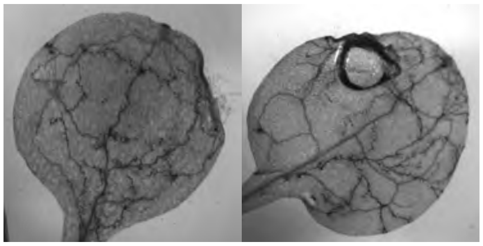

Pna-10

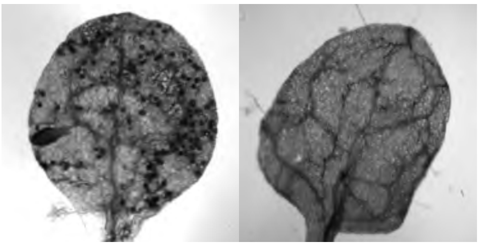

Pro-0

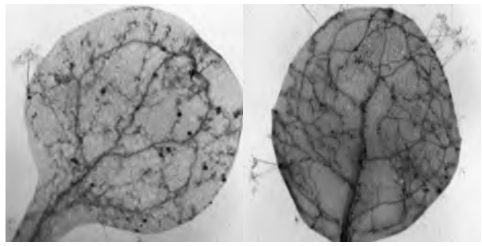

Pu2-23

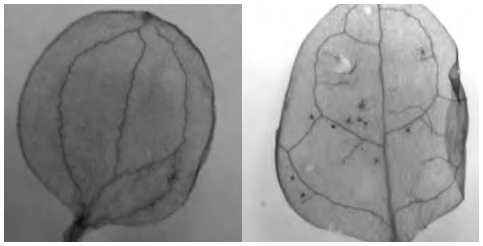

Pu2-7

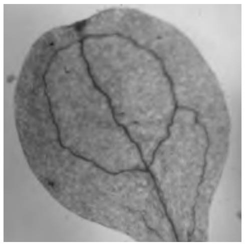

Ra-0

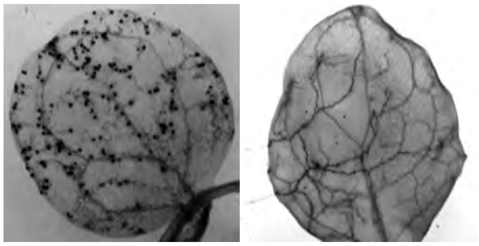

Ren-1

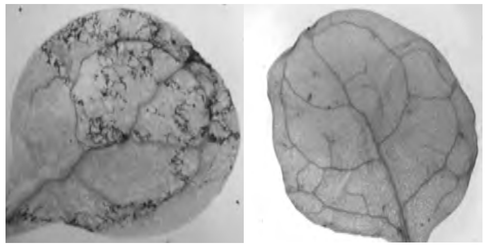

Supplemental Dataset 5. *Hpa* Emwa1

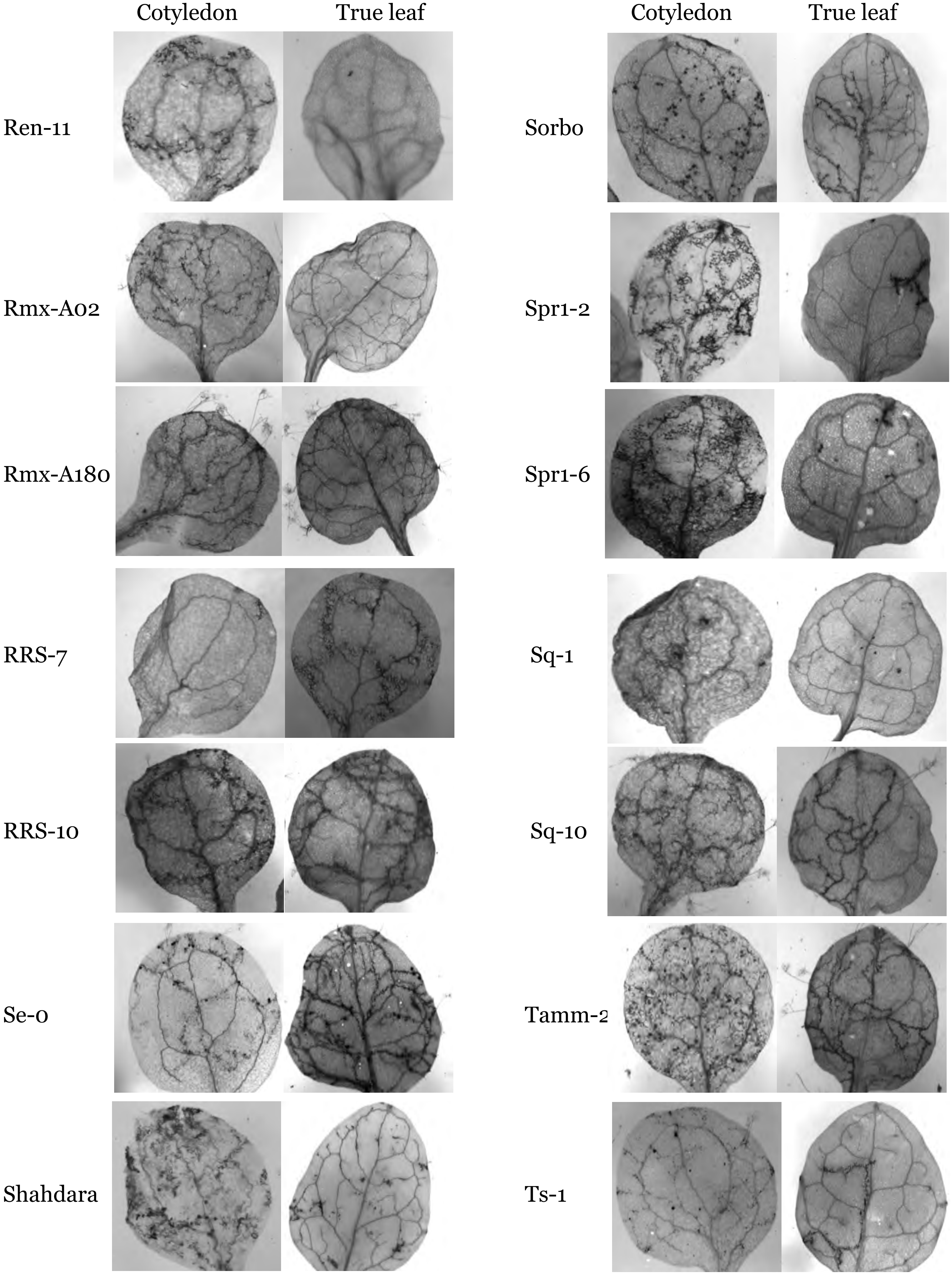

Supplemental Dataset 5. *Hpa* Emwa1

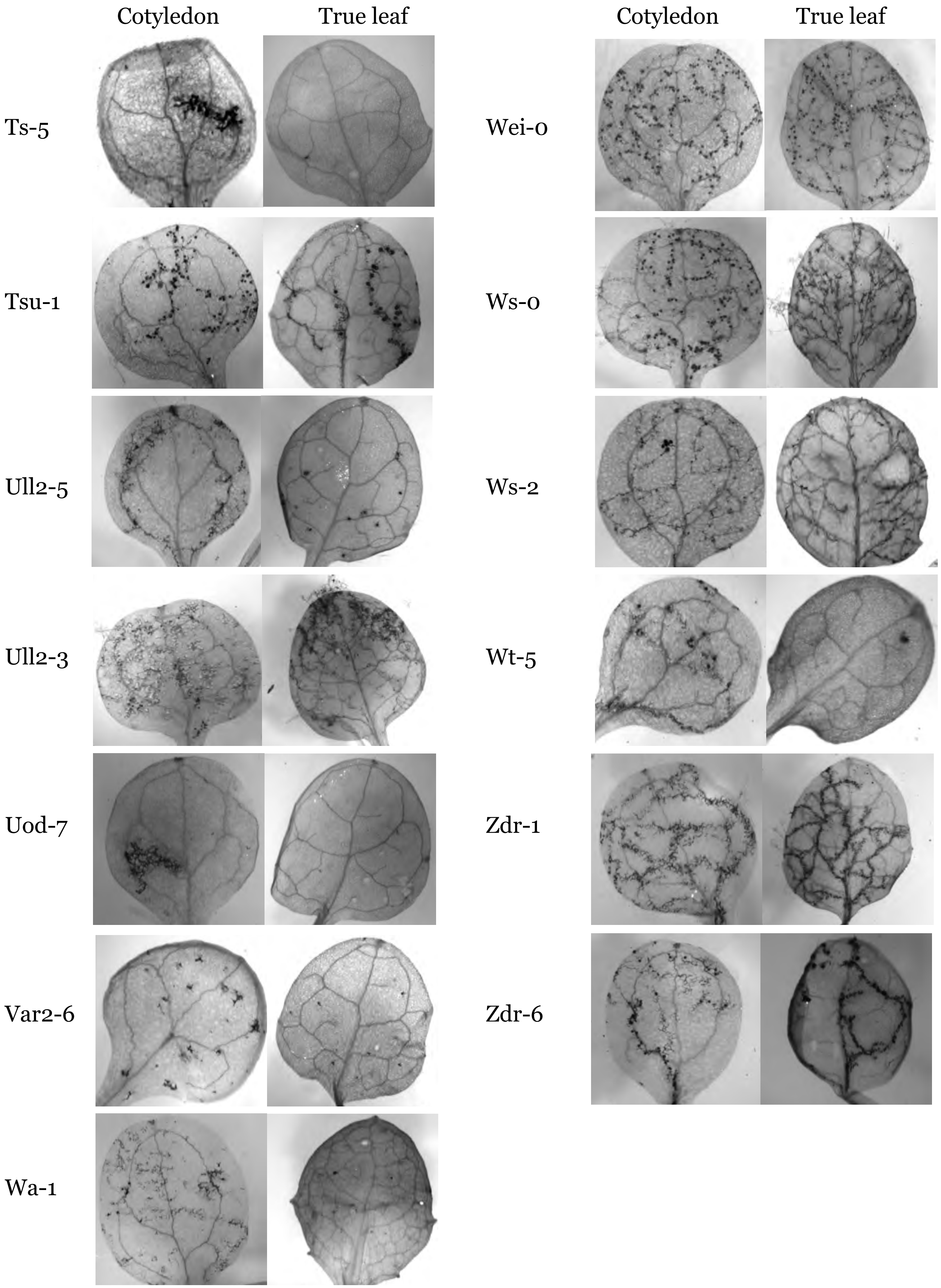

Supplement: Dataset S5 — Images of the trypan blue-stained Arabidopsis cotyledons and true leaves inoculated with Hpa Emwa1. (PDF) [file pone.0028765.s008.pdf]
